# Beneficial Impact of Inhaled 25(OH)-Vitamin D3 and 1,25(OH)2-Vitamin D3 on Pulmonary Response in the Murine Model of Hypersensitivity Pneumonitis

**DOI:** 10.3390/ijms251910289

**Published:** 2024-09-24

**Authors:** Marta Kinga Lemieszek, Michał Chojnacki, Iwona Paśnik, Wiktoria Gawryś, Alicja Wilczyńska, Ilona Leśniowska, Jakub Anisiewicz

**Affiliations:** Department of Medical Biology, Institute of Rural Health, Jaczewskiego 2, 20-090 Lublin, Poland; chojnacki.michal@imw.lublin.pl (M.C.); gawrys.wiktoria@imw.lublin.pl (W.G.); wilczynska.alicja@imw.lublin.pl (A.W.);

**Keywords:** 1,25(OH)2-VD3, 25(OH)-VD3, cholecalciferol, vitamin D3, inhalation, hypersensitivity pneumonitis, extrinsic allergic alveolitis, inflammation, fibrosis

## Abstract

Despite numerous scientific reports on the negative impact of vitamin D3 deficiency on many respiratory diseases, little is known about the influence of this phenomenon on the development and progression of hypersensitivity pneumonitis (HP). The presented study is an attempt to shed light on this occurrence. The research was performed on mouse strain C57BL/6J exposed to the antigen of *Pantoea agglomerans* (etiological factor of HP). To induce vitamin D3 deficiency, mice received a diet with a 10 times lower amount of cholecalciferol than the main control group. VD3-deficient mice inhaled 25(OH)-VD3 or 1,25(OH)2-VD3 used separately or with SE-PA. At the beginning of the experiment and after 14 and 28 days of inhalation, respiratory function was examined using whole-body plethysmography. Moreover, at indicated time points, mice were sacrificed and samples collected for histological examination, flow cytometry, and ELISA. The performed study revealed that inhalations with 25(OH)-VD3 and 1,25(OH)2-VD3 effectively eliminated most of the negative changes in the respiratory system caused by vitamin D3 deficiency by restoring the physiological concentration of 1,25(OH)_2_-VD3 in the body. VD3-deficient mice which inhaled *P. agglomerans* antigen and vitamin D3 metabolites also demonstrated the ability of the tested compounds to eliminate, or at least weaken, the negative effects of the HP causative factor and desired effect, including improvement of respiratory functions and attenuation of inflammation and signs of fibrosis. The obtained results suggested that the beneficial influence of inhaled vitamin D3 metabolites on HP development was associated with the restoration of the physiological concentration of 1,25(OH)2-VD3 in the pulmonary compartments in VD3-deficient mice.

## 1. Introduction

Contrary to popular belief, the term vitamin D3 does not refer to a single substance, but to a group of steroidal fat-soluble compounds, among which we find: (1) cholecalciferol, synthesized in the skin under the influence of sunlight from the 7-dehydrocholesterol, which is the most common supplemented form of vitamin D3, (2) 25(OH)-VD3, also known as 25-hydroxycholecalciferol or 25(OH)-VD3, produced in the liver by hydroxylation of cholecalciferol, the principal circulating form of vitamin D3, and the marker of vitamin D3 level in the body, and (3) 1,25(OH)2-VD3, the biologically active form of vitamin D3, also known as 1,25-dihydroxycholecalciferol or 1,25(OH)2-VD3, mostly produced in the kidneys by hydroxylation of 25(OH)-VD3 [1,2,3].

For decades, vitamin D3 has been associated almost exclusively with regulating the body’s calcium–phosphorus balance and influencing the skeletal system. Research in recent years has proven the pleiotropic effects of vitamin D3, which plays a key role in the proper functioning of the immune, nervous, reproductive, circulatory, respiratory, and musculoskeletal systems [1,2,3,4,5,6]. Although there are two independent ways of obtaining vitamin D3 by animals (endogenous synthesis and food), the prevalence of its deficiency and the resulting diseases have reached the scale of a pandemic [1,6,7].

In recent years, an increasing number of studies have pointed to correlations between vitamin D3 deficiency in the body and the risk of developing and exacerbating many pulmonary diseases, including asthma, cystic fibrosis, chronic obstructive pulmonary disease, and idiopathic pulmonary fibrosis, as well as such respiratory infections such as COVID-19 or tuberculosis [8,9,10,11,12]. Surprisingly little is known about the role of vitamin D3 in the development and progression of hypersensitivity pneumonitis (HP), also known as extrinsic allergic alveolitis (EAA). This heterogeneous group of immunological diseases is caused by chronic exposure to organic dust suspended in the air. In susceptible people, in response to environmental antigens, diffuse inflammatory lesions which develop into granulomas in the lung interstitium and the distal respiratory tract, and in extreme cases, may progress to pulmonary fibrosis [13].

Because worldwide, millions of farmers and workers in many sectors of industry are occupationally exposed to organic dust, the prevalence of HP among indicated groups varies within the range of 1.3% to 12%, while in the case of exposed farmers reached 8% [14,15]. The incidence and prevalence of HP among the worldwide population are variables largely due to seasonal and geographical variability in antigen exposures, lack of internationally accepted diagnostic criteria, and misdiagnosed acute and subacute HP. The last two criteria, as well as common omissions in diagnostics and treatment on the part of people from risk groups, mean that patients do not receive help in the early stages of the disease when the chance for recovery is significant. Unfortunately, most patients are diagnosed in the last stage of diseases with pulmonary fibrosis [16,17]. The most effective therapy in the early stages of the disease is harmful antigen avoidance. In contrast, the effectiveness of therapies with anti-inflammatory (systemic corticosteroids, mycophenolate mofetil, azathioprine, and rituximab) or anti-fibrotic medications (nintedanib and pirfenidone) is controversial. In the case of chronic HP with developed lung fibrosis, the only therapeutical option is lung transplantation [16,17,18,19]. Limited therapeutic options and, in the case of fibrosis, their practical absence constitute an incentive to search for new therapeutic strategies.

Despite the lack of hard scientific evidence of HP connection with vitamin D3 deficiencies, there are many indications for the existence of such a relationship. Among the biological effects of vitamin D3, the most important in the light of potential influence on HP development and progression seem to be as follows: (1) Vitamin D3 impacts respiratory cells, such as alveolar macrophages, airway epithelium, dendritic cells, and lymphocytes T or B-cells, involved in immune responses to environmental antigens including etiological factors of HP [11,20,21,22]; (2) Vitamin D3 plays a pivotal role in the regulation of expression and release of an antimicrobial peptide—cathelicidin—with a proven role in HP development [23,24,25]; and (3) Vitamin D3’s ability to inhibit the epithelial–mesenchymal transition (EMT), primarily by reducing the expression of genes that determine the transformation of epithelial cells into mesenchymal cells, as well as inducing the expression of genes responsible for maintaining the epithelial phenotype and, consequently, preventing the development of pulmonary fibrosis [26,27,28,29].

The purpose of the present study was to enrich the modest resources of knowledge on the role of vitamin D3 in the development and progression of HP. The study was conducted in a murine model of HP [30,31,32], in which pathological changes were induced in the mice strain C57BL/6 prone to fibrosis, by inhalation of the well-known etiological factor of HP—extract of gram-negative bacteria *Pantoea agglomerans* (SE-PA) [33]. Our earlier study revealed that changes provoked by SE-PA are similar to the clinical picture of HP, which was confirmed on the genome and proteome level as well as histological examination of lung tissue and characterization of immune response. Moreover, by modulating the time of animal exposure to SE-PA, successive stages of the disease development are recreated, such as the acute step with a strong inflammatory response (between 7 and 14 days of inhalation), and the chronic step with signs of fibrosis (from the 28th day of inhalation) [30,31,32]. Used for research, the murine HP model reproduces under laboratory conditions the environmental exposure to organic dust leading to the development of acute or chronic HP, dependent on the treatment time. Consequently, its use for these studies increases the clinical relevance of the obtained results. To determine the influence of vitamin D3 on the course of HP, the cholecalciferol content of the animals’ diet was lowered 10-fold, inducing a deficiency of this metabolite in the bodies of the mice. On the other hand, mice on a diet with a standard cholecalciferol content were maintained as the main control of the experiment. Next to the evaluation of the influence of vitamin D3 deficiency on HP development, the presented study focuses on the possibility of using inhaled vitamin D3 metabolites (25(OH)-VD3 and 1,25(OH)2-VD3), administered in doses guaranteeing the restoration of physiological concentration of 1,25(OH)-VD3 in the lung compartments in VD3-deficient mice, to prevent or inhibit inflammation and pulmonary fibrosis in the course of HP. The research focused on the biologically active form of vitamin D3 and its main precursor since our earlier studies proved the efficiency and safety of chronically inhaled 100 pg/g of 25(OH)-VD3 and 5 pg/g of 1,25(OH)2-VD3 in the restoration of physiological calcitriol levels in animals with vitamin D deficiencies [8].

The presented study is based on the following research hypotheses:

1. Vitamin D3 deficiencies negatively impact immune response and fibrosis development in the course of HP;
2. Restoration of the physiological level of calcitriol in lung tissue in VD3-deficient animals prevents or slows down HP development.

It is worth mentioning that the presented study is the first one that addressed the influence of vitamin D3 deficiencies on the course of HP. It is also the first one that examined the direct impact of inhaled 25(OH)-VD3 and 1,25(OH)2-VD3 on the pulmonary response under induced VD3 deficiencies and the course of HP. It is also the first scientific study in which the suggested strategy of HP treatment is based on the restoration of physio-logical pulmonary level of 1,25(OH)2-VD3. Moreover, the presented research is one of few studies that investigated the therapeutical potential of vitamin D3 metabolites delivered to the respiratory tract via inhalations. Although the presented study was performed on animals, and thus, obtained results will require further verification in clinical studies, the above-indicated features prove their uniqueness and exceptionality.

## 2. Results

### 2.1. Restoration of the Physiological Level of 1,25(OH)2-VD3 in Mice with Induced Vitamin D3 Deficiency in Response to Inhalation with Vitamin D3 Metabolites

**Figure 1:**
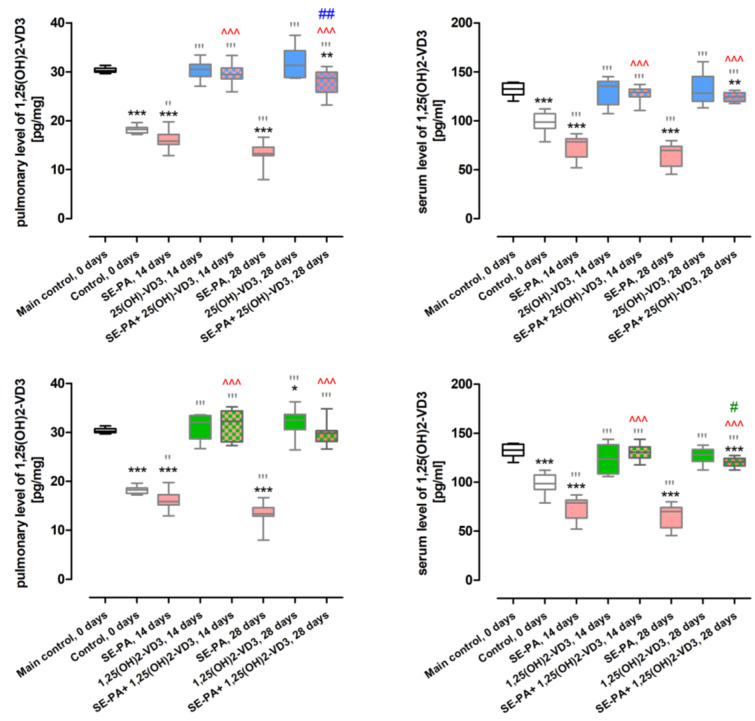
Alterations in pulmonary and serum levels of 1,25(OH)2-VD3 in response to different amounts of cholecalciferol in the diet, inhalation of vitamin D3 metabolites, and exposure to antigen of *Pantoea agglomerans*. Pulmonary and serum concentrations of 1,25(OH)2-VD3 were determined using the ELISA method. Data information: a two-tailed unpaired *t*-test was used for analyses; *p*-value denoted as follows: * *p* < 0.05, ** *p* < 0.01, *** *p* < 0.001 vs. main control; ’’ *p* < 0.01, ’’’ *p* < 0.001 vs. control. Statistically significant differences between data collected at individual time points, from mice exposed to both vitamin D3 metabolites and SE-PA vs. animals treated for indicated times with SE-PA (^^^ *p* < 0.001) or vitamin D3 metabolites (# *p* < 0.05, ## *p* < 0.01) used alone. The numerical data are presented in Supplementary Materials: Table S1.

As presented in Figure 1 and Table S1, mice on the diet with a standard level of cholecalciferol (0.5 IU/g) showed significantly higher concentrations of 1,25(OH)2-VD3 (pulmonary level: 30.31 ± 0.49 pg/mg; serum level: 132.2 ± 6.55 pg/mL) than VD3-deficient mice with the following pulmonary and serum levels, respectively of 1,25(OH)2-VD3: 18.20 ± 0.81 pg/mg and 98.6 ± 9.48 pg/mL.

Mice exposure to saline extract of *Pantoea agglomerans* deepened the 1,25(OH)2-VD3 deficiency induced by the reduced content of cholecalciferol in the diet; pulmonary and serum levels of 1,25(OH)2-VD3 in response to 14 and 28 days of mice treatment with SE-PA were as follows: 15.99 ± 1.96 pg/mg/73.4 ± 11.55 pg/mL and 13.12 ± 2.68 pg/mg/65.7 ± 12.23 pg/mL, respectively.

Animal inhalation of 25(OH)-VD3 at concentrations of 100 pg/g for both 14 and 28 days restored the physiological level of 1,25(OH)2-VD3 in both serum and lung tissue (pulmonary levels: 30.29 ± 1.77 pg/mg and 31.83 ± 3.06 pg/mg; serum levels: 129.2 ± 13.36 pg/mL and 132.1 ± 15.39 pg/mL). On the contrary, mice exposed to 1,25(OH)2-VD3 at the concentration of 5 pg/g effectively restored endogenous serum levels of 1,25(OH)2-VD3 (124.0 ± 14.25 pg/mL and 126.8 ± 7.86 pg/mL), while in the case of pulmonary compartments, this effect was observed only in response to 14 days of treatment (31.08 ± 2.60 pg/mg), while after 28 days of nebulization, the pulmonary concentration of 1,25(OH)2-VD3 was significantly higher than its control level (32.09 ± 2.71 pg/mg).

The short-term exposure of the mice to 25(OH)-VD3 and 1,25(OH)2-VD3 increased the pulmonary and serum levels of 1,25(OH)2-VD3, lowered by SE-PA treatment and deficient diet to physiological amounts: 29.67 ± 2.02 pg/mg/127.7 ± 7.77 pg/mL and 31.46 ± 3.05 pg/mg/130.3 ± 7.90 pg/mL, respectively. Four weeks of VD3-deficient mice inhalation of both SE-PA and vitamin D3 metabolites also significantly elevated pulmonary and serum levels of 1,25(OH)2-VD3 to the following amounts: 27.86 ± 2.72 pg/mg/124.5 ± 4.84 pg/mL (SE-PA + 25(OH)-VD3) and 29.92 ± 2.62 pg/mg/120.3 ± 4.69 pg/mL (SE-PA + 1,25(OH)2-VD3); however, reached values were statistically lower than the physiological.

### 2.2. The Beneficial Impact of Inhalation with Vitamin D3 Metabolites on Respiratory Parameters Altered by Both P. agglomerans Treatment and VD3 Deficiencies

**Figure 2:**
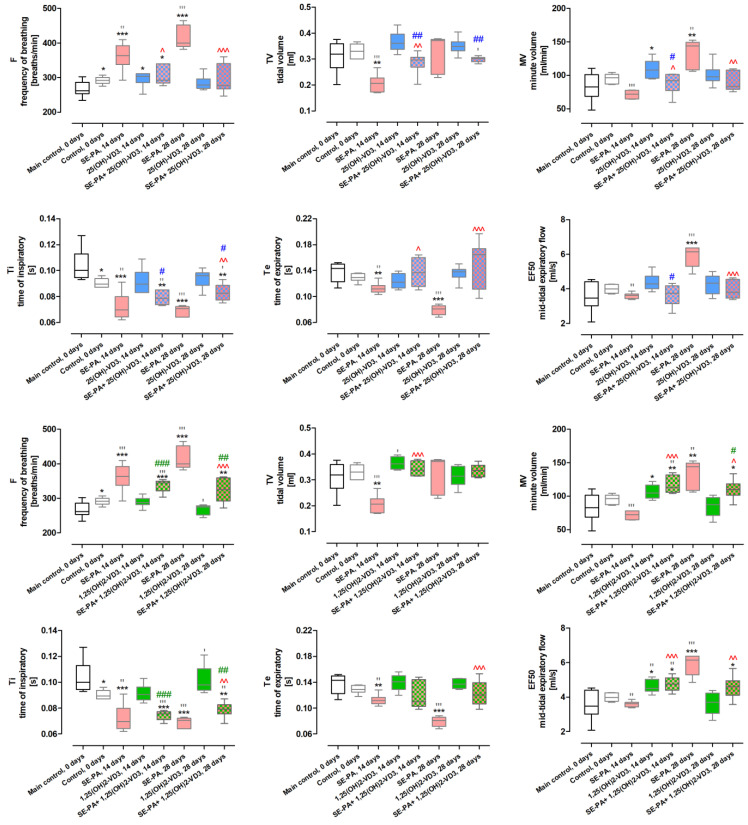
Changes in pulmonary function in VD3-deficient mice exposed to vitamin D3 metabolites and antigen of *Pantoea agglomerans*. Respiratory parameters were investigated using the Non-invasive Airway Mechanics Plethysmograph. The examinations were conducted at the end of every single experiment. Data were collected every 2 s for 10 min. Data information: a two-tailed unpaired *t*-test was used for analyses; *p*-value denoted as follows: * *p* < 0.05, ** *p* < 0.01, *** *p* < 0.001 vs. main control; ’ *p* < 0.05,’’ *p* < 0.01, ’’’ *p* < 0.001 vs. control. Statistically significant differences between data collected at individual time points, from mice exposed to both vitamin D3 metabolites and SE-PA vs. animals treated for indicated times with SE-PA (^ *p* < 0.05, ^^ *p* < 0.01, ^^^ *p* < 0.001) or vitamin D3 metabolites (# *p* < 0.05, ## *p* < 0.01, ### *p* < 0.001) used alone. Numerical data are presented in Supplementary Materials: Table S2.

Whole-body plethysmography (Figure 2 and Table S2) showed that among the six investigated parameters, two (F—frequency of breathing and Ti—inspiratory time) were significantly affected by cholecalciferol diet deficiencies. The respiratory rate (F) recorded in untreated mice in response to cholecalciferol deprivation increased from 266.72 ± 22.97 breaths/min (main control; untreated VD3-sufficient mice) to 291.40 ± 10.79 breaths/min (control; untreated VD3-deficient mice), while inspiratory time (Ti) decreased from 0.104 ± 0.013 s to 0.090 ± 0.004 s under the same conditions.

The negative changes caused by VD3-deficiences intensified in time-dependent manner in the animal response to a saline extract of *P. agglomerans*. The frequencies of breathing recorded after 14 and 28 days of VD3-deficient mice nebulization with SE-PA were 361.13 ± 39.40 breaths/min and 414.28 ± 33.62 breaths/min, while Ti recorded under the indicated conditions reached the following values: 0.072 ± 0.011 s and 0.069 ± 0.004 s. Next to Ti, expiratory time (Te) also revealed similar unfavorable pattern of changes in response to treatment with bacteria antigen; after 14 and 28 days of SE-PA exposure, Te values decreased from 0.112 ± 0.008 s to 0.079 ± 0.008 s. The antigen of *P. agglomerans* also caused in VD3-deficient mice decrease in TV (tidal volume) from 0.310 ± 0.061 mL (main control) to 0.206 ± 0.035 mL (SE-PA,14 days), as well as an increase in EF50 (mid-tidal expiratory flow) from 3.536 ± 0.880 mL (main control) to 5.886 ± 0.618 mL (SE-PA, 28 days).

The respiratory function recorded in VD3-deficient mice after 28 days of treatment with vitamin D3 metabolites did not show any significant changes compared to untreated mice on the standard diet. On the contrary, 14 days of inhalation VD3-deficient mice with 25(OH)-VD3 increased the frequency of breathing (296.47 ± 22.63 breaths/min) and MV-minute volume (109.27 ± 13.87 mL/min). MV and EF50 recorded in VD3-deficient mice after 14 days of treatment with 1,25(OH)2-VD3 were also elevated, reaching the following values: 106.19 ± 10.77 mL/min and 4.621 ± 0.388 mL/s.

25(OH)-VD3 administered together with SE-PA reduced the negative impact of *P*. *agglomerans* extract on pulmonary function of VD3-deficient mice, and the most significant improvement of respiratory functions was recorded after 14 days of 25(OH)-VD3 treatment in the case of TV (0.286 ± 0.043 mL vs. 0.206 ± 0.035 mL) and Te (0.137 ± 0.022 s vs. 0.112 ± 0.008 s), while 28 days of treatment with 25(OH)-VD3 beneficially impacted on F (294.93 ± 42.53 breaths/min vs. 414.28 ± 33.62 breaths/min), MV (89.96 ± 14.74 mL/min vs. 133.84 ± 20.63 mL/min), Te (0.151 ± 0.037 s vs. 0.079 ± 0.008 s), and EF50 (3.930 ± 0.527 mL/s vs. 5.886 ± 0.618 mL/s). Simultaneous administration of 1,25(OH)2-VD3 and *P. agglomerans* antigen significantly inhibited negative changes in MV (109.99 ± 14.87 mL/min vs. 133.84 ± 20.63 mL/min), Ti (0.079 ± 0.006 s vs. 0.069 ± 0.004 s), Te (0.123 ± 0.020 s vs. 0.079 ± 0.008 s), and EF50 (4.569 ± 0.674 mL/s vs. 5.886 ± 0.618 mL/s) caused by 28 days of exposure to SE-PA and VD3-deficient diet. Moreover, 14 days of 1,25(OH)2-VD3 treatment effectively restored only TV (0.342 ± 0.027 mL vs. 0.206 ± 0.035 mL) affected by the antigen and VD3-deficient diet.

### 2.3. The Beneficial Impact of Inhalation of Vitamin D3 Metabolites on Lung Tissue Structure Damaged by Chronic Exposure to the Antigen of P. agglomerans

**Figure 3:**
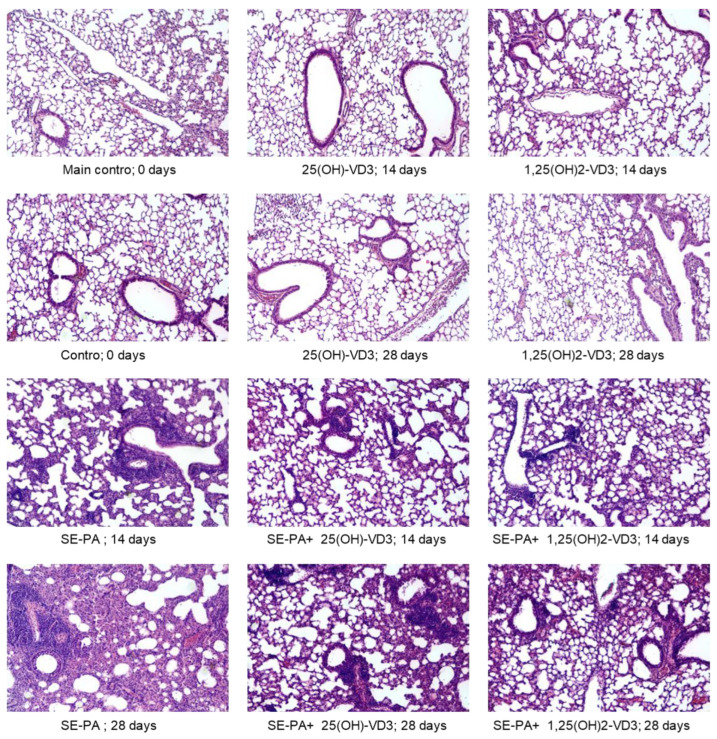
Changes in lung tissue morphology in VD3-deficient mice exposed to vitamin D3 metabolites and antigen of *Pantoea agglomerans*. Lungs collected at the end of each experiment from untreated mice and animals exposed to investigated compounds were stained with hematoxylin and eosin (H&E) and examined under light microscopy at 100× magnification. Representative photographs of mouse lung sections stained with H&E are shown. Numerical data are presented in Supplementary Materials: Table S3.

Light microscopy observation of stained lung samples (Figure 3 and Table S3) revealed discrete changes in the morphology of pulmonary tissues in untreated mice on the deficient diet—focally thickened alveolar septums were observed. Similar types of changes were also observed in response to 14 and 28 days of inhalation of both 25(OH)-VD3 and 1,25(OH)2-VD3. On the contrary, VD3-deficient mice exposure to the antigen of *P*. *agglomerans* for 14 days induced significant centrolobe and interstitial inflammation (granulocytes, mostly neutrophils and reactive pneumocytes, were found), alveolar distortion and thickening of their walls, as well as pulmonary edema (mean score for inflammation was 3.42 ± 0.51; mean score for fibrosis was 2.17 ± 0.39). Signs of fibrosis intensified in response to 28 days of animal inhalation of SE-PA, leading to obstruction of the pulmonary alveoli lumen (mean score for fibrosis was 2.58 ± 0.51), while the inflammatory response remained at a significant level (mean score for inflammation was 3.08 ± 0.29). Negative changes caused by the antigen of *P*. *agglomerans* were reduced by 25(OH)-VD3 and 1,25(OH)2-VD3 treatment; however, the signs of inflammation and focally thickened alveolar septums were still noted. Inflammatory scores for lung tissue collected from VD3-deficient mice after 14 and 28 days of treatment with both SE-PA and 25(OH)-VD3/1,25(OH)2-VD3 were: 1.83 ± 0.39/1.83 ± 0.39 and 1.83 ± 0.39/1.75 ± 0.45. At the same time, fibrosis scores for the above-mentioned research groups were as follows: 1.58 ± 0.51/1.50 ± 0.52 (14 days of inhalation of SE-PA and 25(OH)-VD3/1,25(OH)2-VD3) and 1.67 ± 0.49/1.50 ± 0.52 (28 days of exposure to both SE-PA and 25(OH)-VD3/1,25(OH)2-VD3).

### 2.4. Alterations in Immune Cell Composition in the Course of HP Dependent on Vitamin D3 Inhalations as Well as Cholecalciferol Amounts in the Diet

**Figure 4:**
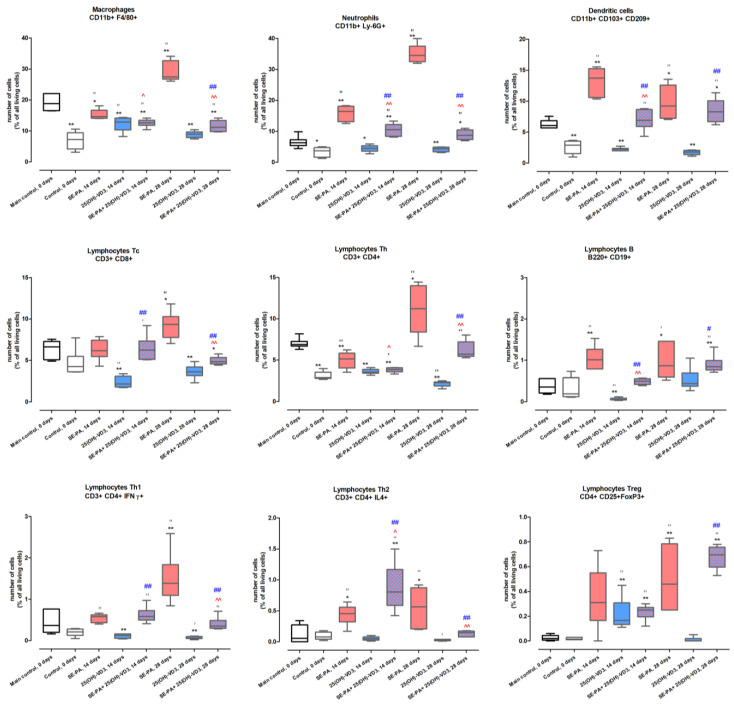

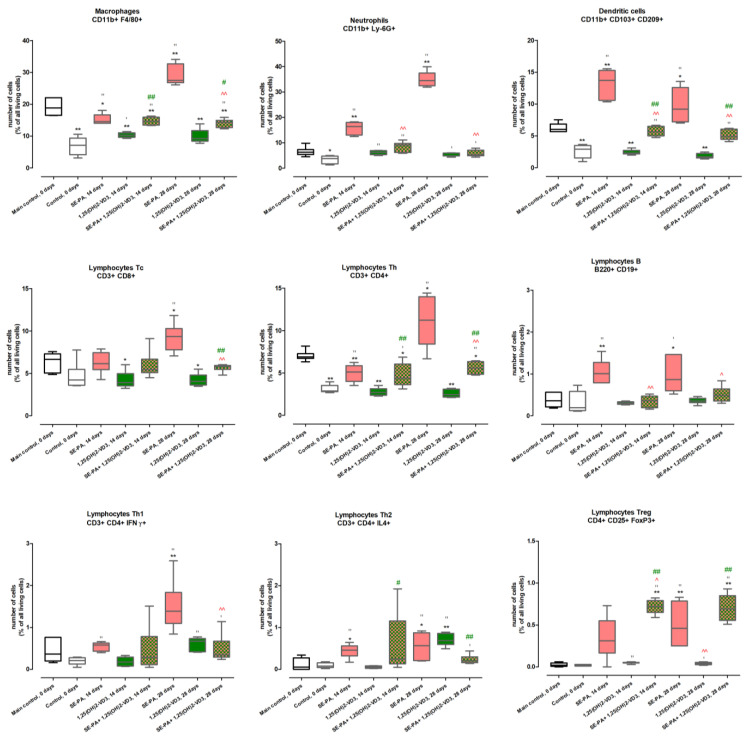
Changes in immune cell composition in VD3-deficient mice exposed to vitamin D3 metabolites and antigen of *Pantoea agglomerans*. Pulmonary cells labeled with antibodies specific for particular markers of immune cells were analyzed using flow cytometry. A minimum of 100,000 cells were acquired and analyzed. Data are presented as percentages of all living cells. Data information: analyses between the two groups were performed using the Mann–Whitney U-test; *p*-value denoted as follows: * *p* < 0.05, ** *p* < 0.01 vs. main control; ’ *p* < 0.05,’’ *p* < 0.01 vs. control. Statistically significant differences between data collected at individual time points, from mice exposed to both vitamin D3 metabolites and SE-PA vs. animals treated for indicated times with SE-PA (^ *p* < 0.05, ^^ *p* < 0.01) or vitamin D3 metabolites (# *p* < 0.05, ## *p* < 0.01) used alone. Numerical data are presented in Supplementary Materials: Table S4.

Results of the flow cytometer (Figure 4 and Table S4) showed that cholecalciferol deprivation in animals’ diet, compared to its standard amount (0.5 IU/g), significantly lowered the level of macrophages (decrease from 18.8% to 7.19%), neutrophils (decrease from 6.27% to 3.71%), dendritic cells (decrease from 6.05% to 2.90%), and lymphocytes Th (decrease from 6.89% to 2.88%). Other investigated immune cells were not affected by cholecalciferol restriction.

The VD3-deficient mice inhalation of SE-PA for 14 days did not impact the percentage of lymphocytes Tc (6.15%) and Treg (0.31%), while at the same time increasing the number of neutrophils (16.37%), dendritic cells (13.72%), B cells (1.01%), and lymphocytes Th2 (0.46%) compared to data collected from untreated mice from both control groups. The percentages of macrophages and lymphocytes Th in VD3-deficient mice incubated with SE-PA for 14 days were at a higher level than recorded in untreated mice on the same diet (14.55% vs. 7.19% and 5.12% vs. 2.88%), while the obtained results were significantly lower than data collected in untreated mice on the standard diet (14.55% vs. 18.80% and 5.12% vs. 6.89%). VD3-deficient mice exposure to the antigen of *P*. *agglomerans* for 28 days elevated the amount of all examined immune cells, compared to untreated mice on diets with normal and reduced amounts of cholecalciferol. Immune cell composition in the mentioned research group was as follows: 34.47% of neutrophils; 27.47% of macrophages; 11.23% of Th (including 1.39% of Th1 and 0.56% of Th2); 9.36% of Tc; 9.20% of dendritic cells; 0.87% of B cells and 0.46% of Treg. The percentage of most of the assessed immune cells in VD3-deficient mice exposed to 25(OH)-VD3 for 14 and 28 days was lower, compared to the main control, including macrophages (12.86% and 8.94%), neutrophils (4.47% and 4.34%), dendritic cells (2.24% and 1.80%), Tc (2.15% and 3.61%), Th (3.52% and 2.28%), and Th1 (0.13% and 0.06%).

Fourteen days of 25(OH)-VD3 nebulization increased the percentage of Treg (0.17% vs. 0.02%), lowered the number of B cells (0.05% vs. 0.35%), and did not change the Th2 level (0.05% vs. 0.06%), while the percentages of indicated cells were on the main control level after 28 days of 25(OH)-VD3 inhalations. Chronic exposure of VD3-deficient mice to 1,25(OH)2-VD3, compared to untreated mice on the standard diet, did not impact the number of neutrophils (6.13% and 5.90%), B cells (0.30% and 0.35%), Th1 (0.18% and 0.67%), and Treg (0.05% and 0.04%). Under the conditions indicated above, the percentage of macrophages, dendritic cells, Tc, and Th cells was lowered, reaching the following values: macrophages, 10.30% and 9.26%; dendritic cells, 2.32 and 1.70%; Tc, 3.93% and 4.07%; and Th, 2.70% and 2.43%. Fourteen days of VD3-deficient mice exposure to 1,25(OH)2-VD3 did not impact the level of Th2, while an additional 14 days of treatment increased this lymphocyte level from 0.06 (14th day of inhalation) to 0.69% (28th day of inhalation).

The administration of 25(OH)-VD3 with SE-PA to VD3-deficient mice revealed the great therapeutic potential of the tested metabolites. Fourteen days of indicated nebulization restored the main control level percentage of dendritic cells (6.91% vs. 6.05%) and B cells (0.50% vs. 0.36%), and came close to the desired values in the case of neutrophils (10.53% vs. 6.27%). Twenty-eight days of VD3-deficient mice inhalation of both 25(OH)-VD3 and SE-PA demonstrated the restoration of the balance disturbed by the mice exposure to bacterial antigen in the case of lymphocytes Th (5.72% vs. 6.89%), Th1 (0.35% vs. 0.37%), and Th2 (0.15% vs. 0.06%). The beneficial impact of 28 days of 25(OH)-VD3 treatment on mice affected by VD3 deficiencies and SE-PA, was also observed in the case of macrophages, neutrophils, and lymphocytes Tc, which reached the levels of 11.12%, 8.68%, and 4.82%, respectively, and strived to restore the balance disturbed by tested harmful agents. Fourteen days of animal inhalation of 1,25(OH)2-VD3 and *P*. *agglomerans* antigen significantly lowered the disturbed by SE-PA percentage of neutrophils (decrease from 16.37% to 9.08%), dendritic cells (decrease from 13.72% to 5.65%), and B-cells (decrease from 1.01% to 0.36%); furthermore, 1,25(OH)2-VD3 treatment restored the percentage of the mentioned cells to the main control levels. Twenty-eight days of mice exposure to 1,25(OH)2-VD3 and SE-PA had a beneficial impact on the number of most of the examined immune cells, except Th2 and Treg. Nevertheless, the greatest results of 28 days of 1,25(OH)2-VD3 treatment, leading to the restoration of the amount of immune cells to the main control level, were observed in the case of neutrophils (decrease from 34.47% to 5.19%), dendritic cells (decrease from 9.20% to 4.95%), Tc (decrease from 9.36% to 5.79%), B-cells (decrease from 0.87% to 0.49%), and Th1 (decrease from 1.39% to 0.35%). The amounts of macrophages and lymphocytes Th elevated by SE-PA were also lowered in response to 1,25(OH)2-VD3 treatment (percentage of macrophages decreased from 27.47% to 14.30%, while the number of Th decreased from 11.23% to 5.57%), and the tested metabolite was not able to completely neutralize the negative impact of bacterial antigen. It should be emphasized that in most cases, animal treatment with vitamin D3 metabolites beneficially influenced or did not impact the immune cell composition changed by exposure to the antigen of *P. agglomerans*. On the contrary, treatment of investigated metabolites may also intensify the effect of SE-PA. The mentioned pattern of changes was observed in Th2 and Treg in response to 14 days of exposure to antigen with 25(OH)-VD3 (increase of Th2 amount from 0.46% to 0.81%) or 1,25(OH)2-VD3 (increase of Treg amount from 0.31% to 0.72%).

### 2.5. Inhibition of Extracellular Matrix Deposition in the Course of HP in VD3-Deficient Mice Caused by Inhalation with Vitamin D3 Metabolites

**Figure 5:**
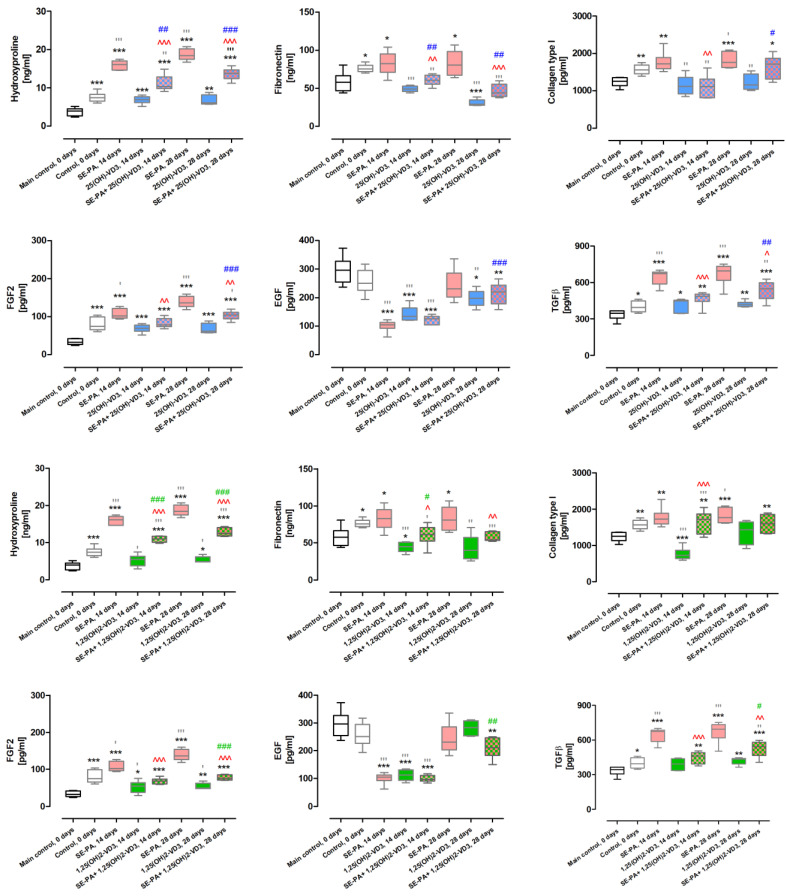
Alterations in extracellular matrix deposition in VD3-deficient mice exposed to vitamin D3 metabolites and antigen of *Pantoea agglomerans*. Protein concentration was examined in lung tissue homogenates using ELISA Kits. Data information: a two-tailed unpaired *t*-test was used for analyses; *p*-value denoted as follows: * *p* < 0.05, ** *p* < 0.01, *** *p* < 0.001 vs. main control; ’ *p* < 0.05, ’’ *p* < 0.01, ’’’ *p* < 0.001 vs. control. Statistically significant differences between data collected at individual time points from mice exposed to both vitamin D3 metabolites and SE-PA vs. animals treated for indicated times with SE-PA (^ *p* < 0.05, ^^ *p* < 0.01, ^^^ *p* < 0.001) or vitamin D3 metabolites (# *p* < 0.05, ## *p* < 0.01, ### *p* < 0.001) used alone. Numerical data are presented in Supplementary Materials: Table S5.

As presented in Figure 5 and Table S5, cholecalciferol deprivation in animal diet, compared to mice on the standard diet, significantly elevated the deposition extracellular matrix indicated by changes in the following concentrations of protein: hydroxyproline (increase from 3.72 ± 1.02 ng/mL to 7.49 ± 1.26 ng/mL), fibronectin (increase from 58.17 ± 13.13 ng/mL to 76.22 ± 5.13 ng/mL), collagen type I (increase from 1.24 ± 0.13 ng/mL to 1.57 ± 0.13 ng/mL), FGF2 (increase from 33.56 ± 8.09 pg/mL to 79.70 ± 17.21 pg/mL), and TGFβ (increase from 334.60 ± 41.95 pg/mL to 400.47 ± 44.90 pg/mL).

VD3-deficient mice exposure to saline extract of *P. agglomerans* elevated the level of indicated proteins, in which concentrations after 14 and 28 days of treatment were as follows: hydroxyproline, 15.97 ± 1.23 ng/mL and 18.68 ± 1.49 ng/mL; fibronectin, 82.67 ± 15.56 ng/mL and 82.61 ± 16.63 ng/mL; collagen type I, 1.78 ± 0.26 ng/mL to 1.82 ± 0.21 ng/mL; FGF2, 106.77 ± 13.49 pg/mL and 138.27 ± 15.03 pg/mL; and TGFβ, 645.14 ± 63.66 pg/mL and 672.21 ± 88.31 pg/mL. Instead, cholecalciferol diet deprivation as well as 14 days of inhalation of SE-PA significantly lowered the concentration of EGF, compared to the main control (100.94 ± 20.13 pg/mL vs. 296.02 ± 47.45 pg/mL).

VD3-deficient mice exposure to 25(OH)-VD3 for 14 and 28 days revealed a decrease in fibronectin (49.03 ± 3.80 ng/mL and 30.91 ± 4.35 ng/mL), collagen type 1 (1.14 ± 0.26 ng/mL to 1.22 ± 0.21 ng/mL), and EGF (142.22 ± 826.18 pg/mL and 197.48 ± 30.96 pg/mL) compared to untreated mice on the same diet. On the contrary, 14 days of animal exposure to 1,25(OH)2-VD3 significantly lowered the amount of four out of six investigated proteins compared to data collected from untreated mice on the same diet (hydroxyproline decreased from 7.49 ± 1.26 ng/mL to 5.15 ± 1.58 ng/mL; fibronectin decreased from 76.22 ± 5.13 ng/mL to 44.26 ± 6.52 ng/mL; collagen type I decreased from 1.57 ± 0.13 ng/mL to 0.76 ± 0.17 ng/mL; and EGF decreased from 256.17 ± 43.38 pg/mL to 111.80 ± 19.24 pg/mL), and this trend in the changes was maintained in the case of hydroxyproline (5.35 ± 0.86 ng/mL), and fibronectin (42.34 ± 17.34 ng/mL) in response to 28 days of inhalation of 1,25(OH)2-VD3. The concentrations of FGF2 and TGFβ were not affected by 25(OH)-VD3 treatment, but significantly increased after 14 and 28 days of inhalation of 1,25(OH)2-VD3, reaching the following values: FGF2, 52.34 ± 15.95 pg/mL and 53.89 ± 8.63 pg/mL; TGFβ, 389.36 ± 53.59 pg/mL and 421.44 ± 32.39 pg/mL.

Both vitamin D3 metabolites administered with the antigen of *P. agglomerans* inhibited the extracellular matrix deposition accelerated by SE-PA. Among recorded changes, the greatest data were noted in the case of fibronectin, whose concentrations after 14 and 28 days of exposure to tested metabolites and SE-PA were maintained at the level of the main control (25(OH)-VD3 + SE-PA: 60.54 ± 7.06 ng/mL and 46.35 ± 8.53 ng/mL; 1,25(OH)2-VD3 +SE-PA: 60.32 ± 13.68 ng/mL and 57.48 ± 6.24 ng/mL). On the contrary, the concentrations of hydroxyproline, FGF2, and TGFβ in VD3-deficient mice treated with both vitamin D3 metabolites and antigen were significantly lower as compared to their levels recorded in animals exposed to SE-PA, but still higher than the concentrations recorded in the main control. After 14 and 28 days of exposure to 25(OH)-VD3 and SE-PA, the concentrations of the above-mentioned proteins were as follows: hydroxyproline, 11.13 ± 2.11 ng/mL and 13.65 ± 1.53 ng/mL; FGF2, 82.51 ± 12.60 pg/mL and 103.16 ± 11.58 pg/mL; and TGFβ, 468.98 ± 61.75 pg/mL and 535.25 ± 78.28 pg/mL. In response to 14 and 28 days of inhalation of 1,25(OH)2-VD3 and Se-PA, concentrations of indicated proteins were as follows: hydroxyproline, 10.87 ± 0.78 ng/mL and 12.76 ± 1.11 ng/mL, FGF2, 68.23 ± 7.88 pg/mL and 77.17 ± 6.69 pg/mL; and TGFβ, 449.14 ± 52.52 pg/mL and 526.66 ± 68.95 pg/mL. In the case of collagen type 1, 14 days of animal treatment with vitamin D3 and SE-PA significantly decreased protein concentration, compared to data obtained from mice exposed to the antigen of *P. agglomerans*; however, only 25(OH)-VD3 was able to restore the main control level of collagen (decrease from 1.77 ± 0.26 ng/mL to 1.11 ± 0.30 ng/mL vs. the main control level: 1.24 ± 0.13 ng/mL).

### 2.6. Changes in the Concentration of Cytokines Involved in Inflammatory and Fibrotic Processes in Response to Vitamin D3 Metabolites Delivered by Nebulization

**Figure 6:**
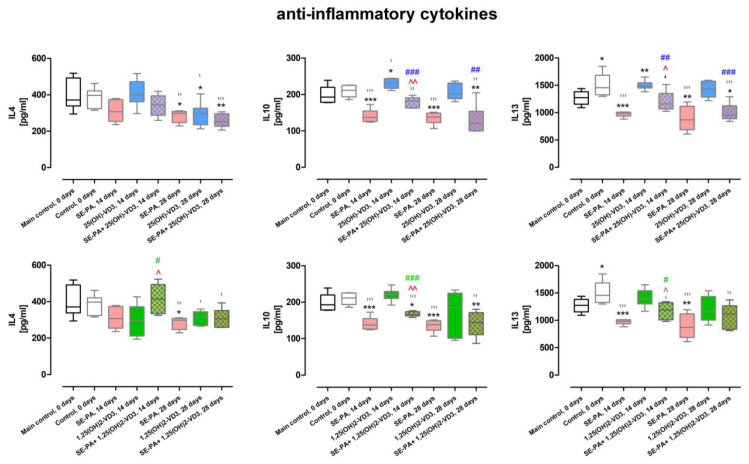

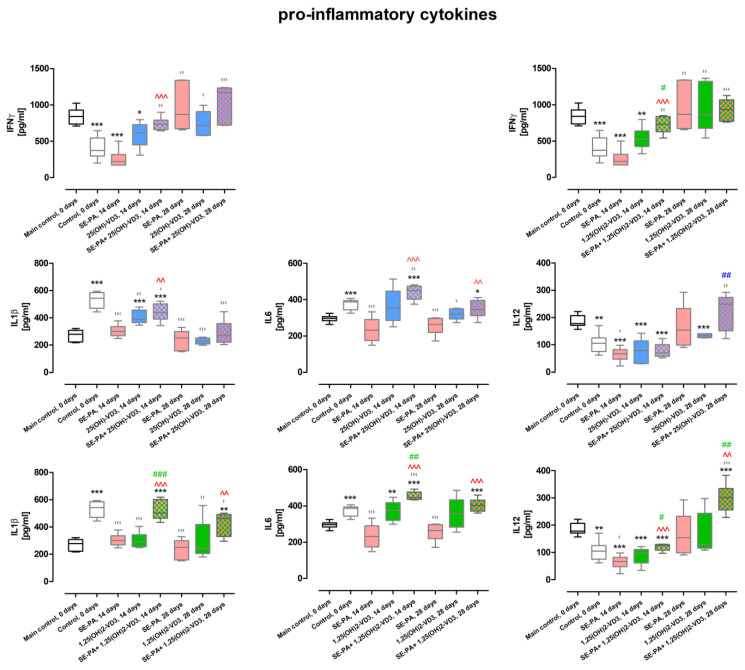
Changes in the concentration of cytokines in VD3-deficient mice exposed to vitamin D3 metabolites and antigen of *Pantoea agglomerans*. Protein concentration was examined in lung tissue homogenates using ELISA Kits. Data information: a two-tailed unpaired *t*-test was used for analyses; *p*-value denoted as follows: * *p* < 0.05, ** *p* < 0.01, *** *p* < 0.001 vs. main control; ’ *p* < 0.05, ’’ *p* < 0.01, ’’’ *p* < 0.001 vs. control. Statistically significant differences between data collected, at individual time points from mice exposed to both vitamin D3 metabolites and SE-PA vs. animals treated for indicated times with SE-PA (^ *p* < 0.05, ^^ *p* < 0.01, ^^^ *p* < 0.001) or vitamin D3 metabolites (# *p* < 0.05, ## *p* < 0.01, ### *p* < 0.001) used alone. Numerical data are presented in Supplementary Materials: Table S6.

As presented in Figure 6 and Table S6, the cholecalciferol deprivation significantly elevated the concentration of IL1β (increase from 270.68 ± 42.25 pg/mL to 530.58 ± 58.95 pg/mL), IL6 (increase from 294.98 ± 19.55 pg/mL to 374.12 ± 30.60 pg/mL), and IL13 (increase from 1268.54 ± 131.28 pg/mL to 1502.71 ± 206.46 pg/mL), and at the same time, lowered the amount of IL12 (decrease from 185.33 ± 22.75 pg/mL to 105.44 ± 37.11 pg/mL) and IFNγ (decrease from 842.28 ± 112.93 pg/mL to 405.01 ± 153.79 pg/mL).

Compared to the main control, VD3-deficient mice exposed to the antigen of *P. agglomerans* for 14 days revealed significantly lowered levels of IFNγ (256.57 ± 124.10 pg/mL), IL10 (140.87 ± 17.86 pg/mL), IL12 (64.20 ± 25.66 pg/mL), and IL13 (970.75 ± 50.11 pg/mL), while after 28 days of exposure, reduced concentration was observed in the case of IL4 (282.48 ± 33.88 pg/mL), IL10 (134.65 ± 16.65 pg/mL), and IL13 (89.97 ± 222.76 pg/mL). It has to be highlighted that among the changes detected in cytokine concentrations in the above-mentioned research groups, typical for SE-PA treatment were modifications in the levels of IL4, IL10, IL12, and IL13 (the same profile of changes was observed in comparison indicated data with both controls). Instead, different pattern of changes in the expression of IFNγ, IL1β, and IL6 suggested that alterations induced by the antigen of *P. agglomerans* dependent on the dietary amount of cholecalciferol. The concentrations of indicated cytokines in the main control, untreated VD3-deficient mice and after 14 and 28 days of VD3-deficient mice inhalation of SE-PA were as follows: IFNγ (842.28 ± 112.93 pg/mL, 405.01 ± 153.79 pg/mL, 256.57 ± 124.10 pg/mL, 958.48 ± 311.17 pg/mL), IL1β (270.68 ± 42.25 pg/mL, 530.58 ± 58.95 pg/mL, 304.10 ± 44.67 pg/mL, 238.70 ± 71.92 pg/mL), and IL6 (294.98 ± 19.55 pg/mL, 374.12 ± 30.60 pg/mL, 233.67 ± 68.09 pg/mL, 254.99 ± 49.01 pg/mL).

Animals given food with a reduced content of cholecalciferol after 14 days of treatment with 25(OH)-VD3 and 1,25(OH)2-VD3, revealed a lowered concentration of IL1 β (403.55 ± 50.29 pg/mL, 302.76 ± 56.44 pg/mL), while the IL10 level increased in response to 25(OH)-VD3 nebulization, reaching the amount of 229.60 ± 13.55 pg/mL. Under the indicated conditions, but after 28 days of exposure to 25(OH)-VD3 and 1,25(OH)2-VD3, the concentrations of IFNγ were higher than in control (744.95 ± 172.58 pg/mL and 972.04 ± 342.65 pg/mL), while the concentrations of IL1β (228.46 ± 22.19 pg/mL and 297.59 ± 149.55 pg/mL) and IL4 (291.98 ± 65.52 pg/mL and 308.18 ± 39.36 pg/mL) were lower than the amounts in untreated mice; moreover, a reduction of the IL6 level was also observed, but only in response to 25(OH)-VD3 (319.18 ± 30.68 pg/mL).

Vitamin D3 metabolite administration together with the antigen of P. agglomerans in-creased the concentration of most cytokines, compared to data collected from animals ex-posed only to SE-PA. The most significant changes were observed on the 14th day of the experiment, during which 1,25(OH)2-VD3 with SE-PA elevated the concentration of all tested molecules, whereas 25(OH)-VD3 with SE-PA affected the concentration of most cy-tokines, with the exception of IL4 and IL12. The concentrations of cytokines elevated by 14 days of 1,25(OH)2-VD3 and 25(OH)-VD3 administered with SE-PA were as follows: IFNγ (724.43 ± 114.61 pg/mL, 737.94 ± 91.64 pg/mL), IL1β (521.11 ± 72.01 pg/mL, 440.07 ± 64.58 pg/mL), IL4 (415.73 ± 82.64 pg/mL), IL6 (453.75 ± 22.58 pg/mL, 439.85 ± 40.23 pg/mL), IL10 (167.17 ± 6.57 pg/mL, 179.09 ± 14.43 pg/mL), and IL12 (119.86 ± 13.21 pg/mL), IL13 (1170.47 ± 158.84 pg/mL, 1205.30 ± 177.30 pg/mL). Among the above-presented changes, highlighted data (bold) shows that the combined administration of vitamin D3 metabolites with P. agglomerans antigen significantly reduced the effect of the SE-PA, and restored cy-tokine levels to the main control level. VD3-deficient mice exposed to vitamin D3 metabo-lites and P. agglomerans antigen for 28 days, compared to mice exposed only to SE-PA, re-vealed elevated levels of IL6 (in response to 25(OH)-VD3 and 1,25(OH)2-VD3; 348.09 ± 48.80 pg/mL, 403.48 ± 35.49 pg/mL) and IL1β and IL12 in response to 1,25(OH)2-VD3 treatment (425.26 ± 85.72 pg/mL, 299.58 ± 352.23 pg/mL).

Vitamin D3 metabolite administration together with the antigen of *P. agglomerans* increased the concentration of most cytokines, compared to data collected from animals exposed only to SE-PA. The most significant changes were observed on the 14th day of the experiment, during which 1,25(OH)2-VD3 with SE-PA elevated the concentration of all tested molecules, whereas 25(OH)-VD3 with SE-PA affected the concentration of most cytokines, with the exception of IL4 and IL12. The concentrations of cytokines elevated by 14 days of 1,25(OH)2-VD3 and 25(OH)-VD3 administered with SE-PA were as follows: IFNγ (724.43 ± 114.61 pg/mL, 737.94 ± 91.64 pg/mL), IL1β (521.11 ± 72.01 pg/mL, 440.07 ± 64.58 pg/mL), IL4 (415.73 ± 82.64 pg/mL), IL6 (453,75 ± 22.58 pg/mL, 439.85 ± 40.23 pg/mL), IL10 (167.17 ± 6.57 pg/mL, 179.09 ± 14.43 pg/mL), and IL12 (119.86 ± 13.21 pg/mL), IL13 (1170.47 ± 158.84 pg/mL, 1205.30 ± 177.30 pg/mL). Among the above-presented changes, highlighted data (bold) shows that the combined administration of vitamin D3 metabolites with *P. agglomerans* antigen significantly reduced the effect of the SE-PA, and restored cytokine levels to the main control level. VD3-deficient mice exposed to vitamin D3 metabolites and *P. agglomerans* antigen for 28 days, compared to mice exposed only to SE-PA, revealed elevated levels of IL6 (in response to 25(OH)-VD3 and 1,25(OH)2-VD3; 348.09 ± 48.80 pg/mL, 403.48 ± 35.49 pg/mL) and IL1β and IL12 in response to 1,25(OH)2-VD3 treatment (425.26 ± 85.72 pg/mL, 299.58 ± 352.23 pg/mL).

## 3. Discussion

Although C57BL/6 is one of the most common animal models for studying pulmonary disorders with signs of fibrosis [34], only some researchers have made an effort to determine the physiological level of vitamin D3 in the bodies of mice and attempted to create a deficiency of this metabolite by modulating the level of cholecalciferol in the animal diet. Our research revealed that the endogenous concentration of 1,25(OH)2-VD3 in 3-month-old male mice strain C57BL/6J on a diet with a cholecalciferol content of 500 IU/kg was 30.31 pg/mg in the lungs and 132.24 pg/mL in the serum. At the same time, the endogenous concentration of 1,25(OH)2-VD3 in mice on a cholecalciferol-deficient diet (50 IU/kg) was 18.20 pg/mg in the pulmonary compartment and 98.61 pg/mL in serum [35]. On the contrary, investigation conducted by Fleet et al. demonstrated that 14-week-old male C57BL/6 mice fed with fodder containing 50 IU/kg and 400 IU/kg of cholecalciferol had the following serum levels of 1,25(OH)2-VD3: 60 pmol/L (almost four times lower than that recorded in our study) and 160 pmol/L (almost twice as low than that recorded in our study in mice on the standard diet) [36]. Nevertheless, another investigation performed by Fleet’s research team on 10-week-old female C57BL/6 mice given a diet with 400 IU/kg of cholecalciferol revealed a serum level of 1,25(OH)2-VD3 around 300 pmol/L, which corresponds to our data [36]. The discrepancies in 1,25(OH)2-VD3 serum levels, noted in the VD3-deficient mice, may have been caused by differences in the length of time the mice remained on a particular diet. In Fleet’s studies, mice were on a dedicated diet for the whole time, whereas in our study, the mice were given standard feed for the first 3 months of life, and only 2 weeks before the beginning of the experiment they were assigned to specific feeding groups, with a strictly controlled amount of cholecalciferol in the diet.

**Figure 7:**
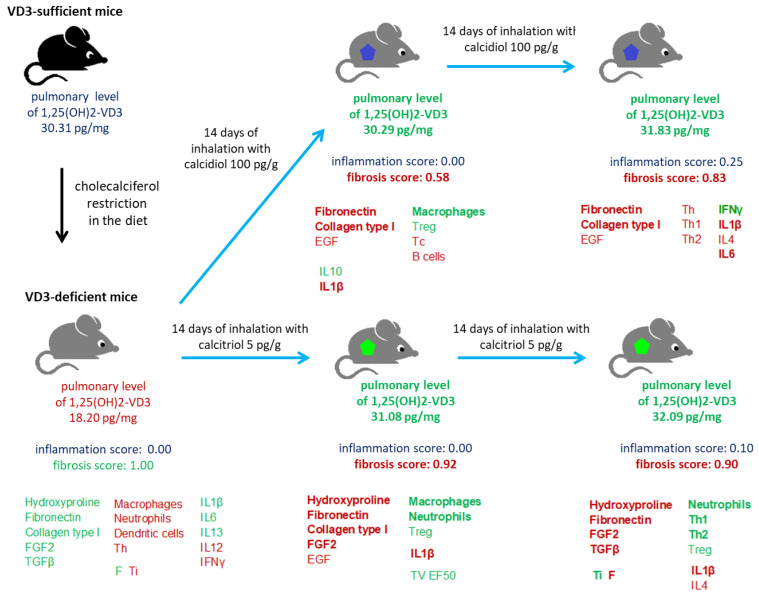
Beneficial impact of inhaled 25(OH)-VD3 and 1,25(OH)2-VD3 on pulmonary function affected by vitamin D3 deficiencies induced by cholecalciferol restriction in mice’s diet. The parameters that increased in response to the analyzed factors (cholecalciferol restriction, 25(OH)-VD3, 1,25(OH)2-VD3) are marked in green, while those that decreased are marked in red. Changes induced by inhalation of vitamin D3 metabolites that were crucial for restoring the disturbed physiological balance and, consequently, for the observed beneficial effects of the proposed therapy are shown in bold. Blue and green pentagons symbolize inhalation with 25(OH)-VD3 and 1,25(OH)2-VD3, respectively.

It needs to be highlighted that our study revealed a high stability of serum levels of 1,25(OH)2-VD3 in both VD3-sufficient and VD3-deficient mice (data collected after 4 and 6 weeks from the beginning of animal feeding with a controlled cholecalciferol content), which indicated the usefulness of creating feeding models. The serum concentrations of 1,25(OH)2-VD3 at the indicated time points in the case of the standard diet were 132.53 pg/mL and 134.73 pg/mL, while in the case of the deficit diet, they were 97.51 pg/mL and 97.76 pg/mL (Figure 7). It is also worth emphasizing that our team was the first to assess the endogenous level of 1,25(OH)2-VD3 in murine lungs, proving that cholecalciferol deficiency in the animals’ diet directly impacts on this metabolite amount in the respiratory system.

Histological analysis revealed focal septal thickening in lung tissue of untreated VD3-deficient mice compared to VD3-sufficent animals. Discovered changes in lung morphology corresponded with the significant increase of hydroxyproline, fibronectin, collagen type 1, FGF2, and TGFβ in untreated mice with reduced serum and pulmonary levels of 1,25(OH)2-VD3. At the same time, results of whole-body plethysmography showed deterioration of respiratory parameters (increased frequency of breathing and decreased inspiratory time) in VD3-deficient mice (Figure 7). The obtained data correspond with results collected by Shi et al., who investigated the influence of chronic vitamin D3 deficiencies in fibrosis development on the offspring of the VD3-deficient C57BL/6J mice remaining on a reduced diet throughout the experiment. After 2 months of animals feeding on a cholecalciferol-deficient diet, they reported disrupted alveolar structure: the alveoli were irregular, distorted, or even collapsed. Changes in lung tissue morphology were associated with elevated levels of hydroxyproline, fibronectin, collagen type I, α-smooth muscle actin, and TGFβ1, which all together indicated increased extracellular matrix deposition [37]. In light of our plethysmography data, it is worth mentioning the results obtained by Zosky et al. in the offspring of the VD3-deficient BALB/c mice that stayed on a deficient diet for 2 weeks before the examination. They reported, for the first time, a significant shortage in the respiratory function in the VD3-deficient mice associated with the decrease in lung volume, as well as overall lung size [38]. Additional studies conducted in the above-mentioned research models confirmed an earlier reported negative correlation between vitamin D3 status and lung function, and also provided new observations. Nunez et al. discovered that 8-week-old male and female whole-life VD3-deficient BALB/c mice had altered pulmonary function, particular parenchymal tissue mechanics. They observed an increase in H value (tissue elastance; the measure of tissue stiffness) and G value (tissue damping; lung mechanics parameter linked to the small airways and ventilation heterogeneity). Moreover, they also reported a significant accumulation of eosinophils and lymphocytes (the type of lymphocytes was not indicated) in bronchoalveolar lavage (BAL) collected from whole-life VD3-deficient BALB/c mice. Nevertheless, despite the strong impact of vitamin D3 deficiency on the pressure-dependent parenchymal mechanics, as well as immune cell number, they did not report any changes in pulmonary expression of *COL1A1* gene, coding the Collagen type 1 [39]. In light of the immune response, our study revealed a negative correlation between pulmonary concentration of 1,25(OH)2-VD3 and the amount of macrophages, neutrophils, dendritic cells, and lymphocytes Th in lung tissue. Cholecalciferol restriction also affected the pulmonary cytokine profiles, increasing the levels of IL1β, IL6, and IL13, while at the same time lowering the levels of IFNγ and IL12. In contrast to our study, research conducted by Seldeen et al. on 6-month-old male C57BL/6J mice consuming a diet with 1000 IU/kg vitamin D3 or 125 IU/kg vitamin D3 for 12 months revealed a lack of significant differences in the serum levels of several investigated cytokines, including IL1α, IL1β, IL6, IL10, IL15, IL18, MCP, and TNFα. The lack of significance could be due to the lack of statistically significant changes in serum concentration of 1,25(OH)2-VD3 between the indicated nutritional groups (1,25(OH)2-VD3 concentration around 90 pg/mL), despite evident differences in the serum level of 25(OH)-VD3 in VD3-deficient mice (25(OH)-VD3 level on deficient diet was in the range of 10–15 ng/mL, while on the standard diet was in the range 0f 30–40 ng/mL) [40]. The cited data indicate the limitations of using measurements of serum concentration of 25(OH)-VD3 to predict the level and biological activity of vitamin D3 in the body and, consequently, to predict health consequences, and if required, to plan therapeutic activities.

Our earlier studies conducted on 3-month-old male mice strain C57BL/6J receiving a standard diet have shown that animals after 14 days of exposure to saline extract of *P. agglomerans* revealed significant inflammatory responses (histological mean score for inflammation was 1.60/2.11*) associated with the accumulation of macrophages, lymphocytes B, Th, and Treg, as well as increased concentrations of IL1, IL4, IL5, IL10, IL12, IL13, IFNγ, TNFα, and TGFβ1. Moreover, the first signs of fibrosis, including thickening of alveolar walls (histological mean score for inflammation was 0.80/1.06*), as well as increased deposition of hydroxyproline and collagens types 1, 2, and 3, were also noted. All mentioned changes intensified in response to 28 days of mice inhalation of SE-PA (histological mean score for inflammation and fibrosis reached the following values: 2.00/2.64* and 1.50/1.98*). Moreover, pulmonary function tests revealed a significant worsening of breathing frequency and lung total volume during mice treated with the antigen of P. agglomerans. *In a previous study, a four-point scale was used to assess histological changes; the results were converted to the five-point scale used in this study, and displayed in bold [25,30]. In the presented study (Figure 7), 3-month-old male mice strain C57BL/6J with induced VD3 deficiencies in response to chronic exposure to SE-PA also developed significant inflammatory as well as fibrotic responses, which intensified during the time of treatment; nevertheless, the range and character of changes were different. After 14 days of treatment with SE-PA, inflammatory scores reached 3.42, which was associated with the accumulation of neutrophils, dendritic cells, lymphocytes B, and Th2, simultaneously compared to the main control. Moreover, significant decrease in concentrations of IFNγ, IL10, IL12, and IL13 was also noted. Regarding fibrosis, performed studies revealed evident extracellular matrix deposition related to elevated levels of hydroxyproline, fibronectin, collagen type 1, and increased concentrations of associated growth factors FGF2 and TGFβ. Histological examination assessed the fibrotic changes for 2.17 scores. Whole-body plethysmography demonstrated a worsening of respiratory function, including increase in breathing frequency and decrease in TV, Ti, and Te. After 28 days of VD3-deficient mice inhalation of SE-PA, the described changes intensified; histological scores for inflammation reached 3.08 points, which was associated with lowered levels of anti-inflammatory cytokines IL4, IL10, and IL13, as well as increased levels of all investigated immune cells (macrophages, neutrophils, dendritic cells, lymphocytes Tc, Th1, Th2, B, and Treg). Microscopic observations revealed additional thickening of the alveoli walls, leading to obstruction of their lumen (fibrosis mean score was 2.58). The mentioned changes were supported by an additional increase in fibrotic markers: hydroxyproline, fibronectin, collagen type 1, FGF2 and TGFβ. Devastation of respiratory function also accelerated, and next to the previously mentioned changes, a significant increase in indicator of bronchoconstriction (EF50) was observed.

Although the simplest and the most efficient way to maintain the proper level of vitamin D3 in the body is to enable its endogenous synthesis by skin exposure to the sun, there has been increased public awareness about the pro-carcinogenic and pro-aging effects of exposure to UVB radiation [1,41,42]. Consequently, most strategies for eliminating vitamin D3 deficiencies are based on the supplementation of its metabolites [1,43]. However, as numerous studies have shown, including those assessing the possibility of using vitamin D3 supplements in the prevention and treatment of lung diseases, the effectiveness of this strategy is questionable [1,8,10,44]. The main reasons for the ineffectiveness of therapeutic strategies based on vitamin D supplementation were comorbidities that the investigated patients suffered and metabolic disorders that altered the synthesis of 1,25(OH)2-VD3 [8,10]. Moreover, inappropriate supplementation is associated with the danger of vitamin D3 overdose, leading to hypercalciuria, hypercalcemia, and hyperphosphatemia, which, if left untreated, can cause kidney stones, calcification of blood vessels, and organ parenchyma [1]. The danger of systemic toxicity following oral or parenteral administration of too high doses of classical vitamin D metabolites is easier to understand when we realize that the transformation of 25(OH)-VD3 to 1,25(OH)2-VD3 occurs not only in kidneys, but also in many other body compartments [2,45]. Either locally produced or directly delivered 1,25(OH)2-VD3, after binding with the vitamin D receptor (VDR) presented in almost every nucleated cell in our body, regulates the expression of over 200 different genes [2,45,46]. To avoid the above-mentioned undesirable side-effects, vitamin D metabolites with reduced toxicity are being developed, e.g., CYP11A1-derived 20(OH)-VD3, 20(OH)-VD2 and 20,23(OH)2-VD3, which are not toxic and non-calcemic at very high doses (3–60 µg/kg), in contrast to 25(OH)-VD3 and 1,25(OH)2-VD3 [47,48,49]. Another way to reduce the risk of systemic toxicity is direct delivery of 25(OH)-VD3 and/or 1,25(OH)2-VD3 to the respiratory system in order to obtain a beneficial pro-health effect without uncontrolled activation of vitamin-D-target genes in other body compartments [35,47,50,51,52,53,54]. Although the concept of directly administering vitamin D3 metabolites to the respiratory tract in order to prevent or treat pulmonary diseases associated with vitamin D3 deficiencies is not new, our team went a step further by focusing on restoring the physiological concentration of 1,25(OH)2-VD3 in the respiratory tract [35]. The mentioned study revealed that restoration of the physiological levels of 1,25(OH)2-VD3 in VD3-deficient mice required 7 days of exposure to 100 pg/g of 25(OH)-VD3 or 5 pg/g of 1,25(OH)2-VD3. Moreover, the indicated interventions did not cause any negative changes in mice’s respiratory functions [35]. The presented studies also demonstrated the effectiveness of 14 and 28 days of VD3-deficient mice inhalation of 100 pg/g of 25(OH)-VD3 or 5 pg/g of 1,25(OH)2-VD3 in recovery physiological 1,25(OH)2-VD3 concentrations; however, on the 28th day of 1,25(OH)2-VD3 inhalation, the pulmonary concentration of 1,25(OH)2-VD3 was higher than the main control.

Histological examination revealed that the indicated intervention did not cause any statistically significant changes in lung tissue morphology in the light of inflammatory responses and signs of fibrosis, compared to untreated mice on both VD3-sufficient and VD3-deficient diets. On the contrary, some changes in respiratory response to nebulization with vitamin D3 metabolites were observed in other investigations. Whole-body plethysmography revealed discrete changes after 14 days of mice treatment with both tested metabolites. Compared to the main control, 25(OH)-VD3 increased the frequency of breathing and MV, while 1,25(OH)2-VD3 elevated values of EF50 and MV. It needs to be highlighted that 28 days of mice exposure to the tested metabolites did not show any negative alterations in animal’s respiratory function; moreover, 1,25(OH)2-VD3 effectively neutralized the negative impact of cholecalciferol deprivation on the frequency of breathing and Ti.

The beneficial effect of inhalation of D3 metabolites was also observed in the area of extracellular matrix proteins, the level of which significantly increased in response to vitamin D3 deficiency. 25(OH)-VD3 significantly reduced fibronectin and collagen type 1 levels, while 1,25(OH)2-VD3 positively influenced all investigated ECM markers. It is worth emphasizing that 28 days of 25(OH)-VD3 inhalation lowered fibronectin levels below the value observed in the main control. Instead, 14 days of mice exposure to 1,25(OH)2-VD3 decreased fibronectin and collagen type 1 concentrations below the main control levels. Investigated metabolites also had a favorable influence on FGF2 and EGF factors. 25(OH)-VD3 inhalation caused a decrease in EGF concentration below the values observed in the controls. 1,25(OH)2-VD3 significantly reduced the level of FGF2 increased by vitamin D3 deficiency. At the same time, 1,25(OH)2-VD3 lowered the EGF concentration in mice exposed to it for 14 days. Simultaneously, the tested metabolites did not affect the level of TGFβ in animals with vitamin D3 deficiency. The discovered beneficial influences of investigated metabolites on the key components, as well as modulators of extracellular matrix deposition, mainly by maintaining the physiological levels of the mentioned factors or reducing undesirable changes caused by vitamin D3 deficiency, correlated with the above-described changes in the lung tissue in terms of signs of fibrosis, suggesting the antifibrotic potential of the examined compounds.

Although histological evaluation did not show any signs of inflammation in lung tissue of VD3-deficient mice exposed to 25(OH)-VD3 and 1,25(OH)2-VD3, alterations in immune cell composition as well as production and release of several cytokines were observed. Nevertheless, a detailed analysis of the results showed that the tested metabolites reduced or did not affect the physiological number of immunocompetent cells in the lung tissue, except for an increased number of Treg lymphocytes in mice exposed to 25(OH)-VD3 for 14 days, and an increased percentage of Th2 lymphocytes in response to a 28-day exposure to 1,25(OH)2-VD3. The indicated changes were anti-inflammatory features, which coincide with the microscopic image. Changes in the cytokine profile induced by nebulization with vitamin D3 metabolites also contributed to the suppression of the inflammatory response, as evidenced by a decrease in the level of pro-inflammatory cytokines IFNγ and IL12 in response to both metabolites, and an increase in the level of anti-inflammatory IL10 and IL13 in response to 25(OH)-VD3. Derogation of this feature was elevated levels of IL1β and IL6 noted after 14 days of exposure to 25(OH)-VD3 and 1,25(OH)2-VD3, respectively. Nevertheless, it has to be emphasized that increased levels of pro-inflammatory IL1β and IL6 were lower than noted in untreated VD3-deficient mice. In the context of vitamin D3 deficiency, it is worth mentioning that inhalations with vitamin D3 metabolites have proven to be effective in restoring the immune balance disturbed by a cholecalciferol deficit in the diet. This was particularly noticeable in the case of 1,25(OH)2-VD3 inhalation, which effectively eliminated the effect of cholecalciferol deprivation on the level of neutrophils, macrophages, and the amount of IL1 and IFNγ. In the case of 25(OH)-VD3, this beneficial effect was observed in the case of macrophages and the cytokines IL1, IL6, and IFNγ.

Unfortunately, there is a deficit of data about the influence of vitamin D3 inhalations on the respiratory response, which we could compare with the results of our study. Due to the analyzed metabolites (metabolites dissolved in PBS, 25(OH)-VD3 at concentrations of 100, 500, and 1000 pg/g; 1,25(OH)2-VD3 at concentrations of 1, 10, and 50 pg/g) and the time of nebulization (14 days), it is worth mentioning Taylor’s research [54]. Taylor et al. reported the beneficial influence of 25(OH)-VD3 and 1,25(OH)2-VD3 nebulization on rat neonatal lung maturation supported by elevated surfactant production, enhancement of alveogenesis, and increase in differentiation of endothelial cells, lipofibroblasts, and alveolar type II cells. The discovery of the advantage impact of vitamin D3 metabolites on lung tissue was observed in studies similar to ours, regarding study times of treatment as well as compound’s concentrations. Furthermore, both studies confirmed the higher efficiency of 1,25(OH)2-VD3 treatment, which in much lower doses than 25(OH)-VD3, revealed similar beneficial effects [54]. Of course, this last observation was not surprising in light of the bioactivities of tested compounds. Nevertheless, it provides valuable guidance on therapeutic dosing of the mentioned metabolites in future translational studies. It is also worth mentioning the vitamin D3 levels assessed during the discussed experiment. As previously mentioned, the results of our studies showed the restoration of physiological concentrations of 1,25(OH)2-VD3 in the lung tissue and serum of VD3-deficient mice in response to 14 days of inhalation of 25(OH)-VD3 or 1,25(OH)2-VD3. At the same time, Taylor’s research demonstrated a lack of changes in serum levels of 25(OH)-VD3 and calcium after 14 days of inhalation of vitamin D3 metabolites in relation to untreated rats [54]. For obvious reasons, although these data cannot be compared, they suggest the safety of the proposed therapeutic strategy. The usefulness of 1,25(OH)2-VD3 inhalations in the treatment of pulmonary diseases also supported results obtained by Horiguchi et al., who revealed alveolar regeneration in a mouse model of chronic obstructive pulmonary disease in response to 1,25(OH)2-VD3. Intrapulmonary administration of 1,25(OH)2-VD3 (10, 100, 1000 pg/g) twice a week for 2 weeks, significantly shortened the distance between alveolar walls, recovered tissue elastance and respiratory competence (FEV 0.05/FVC; forced expiratory volume in 0.05 s to the forced vital capacity) in elastase-induced emphysema [53]. Alveolar repair in response to 1,25(OH)2-VD3 treatment was also reported in another murine model of COPD with progressive pulmonary emphysema. An adiponectin-deficient 50-week-old male mouse which received transpulmonary twice a week for 30 weeks, 1,25(OH)2-VD3 at a concentration of 1000 pg/g, revealed a significant reduction in the distance between alveolar walls as well as improvement of other disease symptoms, including body weight and bone density [52].

The beneficial effect of inhalation of vitamin D3 metabolites was also observed in the reduction of negative changes caused by mice exposure to saline extract of *P. agglomerans.* Comparison data collected from animals treated with SE-PA with results obtained from mice exposed to both SE-PA and vitamin D3 metabolites revealed the mitigation of respiratory disturbances caused by antigen. Almost all the analyzed respiratory parameters, which changed significantly in response to SE-PA compared to the main control, tended to equalize when the antigen was administered together with the tested metabolites, and in the case of 28 days of 25(OH)-VD3 therapy, almost all parameters (except Ti) returned to the levels observed in untreated VD3-sufficient mice. Next to the advantageous influence of vitamin D3 treatment, the disturbances in MV and EF50 levels recorded in mice exposed for 14 days to SE-PA and 1,25(OH)2-VD3 needs to be mentioned. Importantly, the level of the indicated respiratory parameters in response to the antigen itself did not change compared to the main control; however, MV and EF50 values were elevated in response to the tested metabolites used alone, and remained at this increased level in animals exposed to both SE-PA and 1,25(OH)2-VD3. Nevertheless, mice exposed to 28 days of the *P. agglomerans* antigen increased the indicated parameters, unlike 1,25(OH)2-VD3 nebulization, where the MV and EF50 values were at the control level, while the combined administration of the SE-PA and 1,25(OH)2-VD3 influenced them beneficially. Histological analysis also showed a beneficial effect of vitamin D3 metabolite therapy on changes caused by cholecalciferol deficiency and *P. agglomerans* antigen. However, as far as a clear reduction in inflammation was observed in mice exposed to SE-PA and the tested metabolites, compared to animals exposed to the antigen alone, in the case of signs of fibrosis the beneficial effect was noticeable, but not as spectacular. This was reflected in the results of other biochemical analyses. Flow cytometry showed that the combined administration of SE-PA and vitamin D3 metabolites reduced the number of immunocompetent cells elevated by VD3 deprivation and SE-PA inhalations. 1,25(OH)2-VD3 therapy proved to be particularly effective in this respect; 1,25(OH)2-VD3 administered with SE-PA reinstated to the main control level the percentage of neutrophils, dendritic cells, Tc, B, Th1, Th2, and Treg, the amount of which was raised by the antigen. It is also worth mentioning the alterations in the percentage of macrophages, whose representation in the lungs of VD3-deficient mice exposed to antigen and tested metabolites, decreased below the values recorded in the main control. Next to the reduction in the total percentage of pulmonary immune cells, the suppression of the inflammatory response observed in the above-described microscopy images, is also supported by the increase in the level of anti-inflammatory lymphocytes Th2 (25(OH)-VD3) and Treg (1,25(OH)2-VD3) in mice exposed for 14 days to the *P. agglomerans* antigen and tested metabolites. Analysis of the cytokine profiles also showed that the beneficial effect of therapy based on vitamin D3 metabolites is closely related to the restoration of the immunological balance disturbed by diet cholecalciferol deprivation and exposure to SE-PA. The tested metabolites increased or maintained unchanged the concentration of cytokines, the level of which was reduced as a result of exposure to the above-indicated harmful agents. Moreover, in each of these cases, the concentration of cytokines increased to the level recorded in the main control. It is worth mentioning the effect of the combined administration of the *P. agglomerans* antigen and the tested metabolites on the pro-inflammatory IL1B and IL6, the concentration of which in VD3-deficient animals exposed to the SE-PA alone did not change compared to the main control, but increased in response to metabolite therapy. Surprisingly, the discussed research groups showed a strong decrease in the percentage of macrophages, which are the main source of IL1B and IL6. Moreover, alternative sources of IL1 (dendritic cells) and IL6 (B lymphocytes) also seem unlikely because the slight increase of dendritic cells and B cells was only observed on day 28 of 25(OH)-VD3 therapy, while in the considered variants, they were at the main control levels. Analysis of the impact of vitamin D3 metabolite therapy on the concentration of extracellular matrix components and factors modulating ECM deposition once again demonstrated the tendency of the tested metabolites to restore the balance disturbed by the *P. agglomerans* antigen and cholecalciferol deprivation, and in the case of fibronectin, a complete neutralization of the negative effects of the indicated harmful factors was observed. Although on the 14th day of the experiment in VD3-deficient mice inhaled simultaneously with SE-PA and tested metabolites, physiological concentrations of 1,25(OH)2-VD3 were observed both in the lungs and in the serum. The above-mentioned data showed that inhalations with VD3 metabolites are not able to neutralize all negative changes caused by *P. agglomerans* antigen and cholecalciferol deprivation. On the other hand, the fact that on the 28th day of combined administration of SE-PA and VD3 metabolites, the physiological concentration of 1,25(OH)2-VD3 in VD3-deficient mice was restored only in the lungs of animals treated with 1,25(OH)2-VD3 was also not reflected in the results presented above. The obtained results did not indicate a greater effectiveness of 1,25(OH)2-VD3 inhalation over 25(OH)-VD3 inhalation in alleviating, or even counteracting, the negative consequences of induced vitamin D3 deficiency or inhalation of the etiological HP agent.

As mentioned before, only a few scientific reports present the impact of vitamin D3 nebulization on the respiratory system response under pathological conditions. One such study is the Reddy et al. investigation, which caught our attention mostly because of the concept of the study, based on the chronic exposure of Swiss albino mice infected with *Mycobacterium tuberculosis* (Mtb) to 1,25(OH)2-VD3 at a concentration of 5 pg/g. The mice were exposed to 1,25(OH)2-VD3 once daily, 5 days a week, for 28 days. Unfortunately, the level of vitamin D3 in the bodies of the tested animals was not determined. Despite the fact that the examined intervention did not inhibit the MTb burden or intensify of the production of immune peptide (cathelicidin), it revealed inhibition of inflammation, especially mononuclear cell infiltration, less fibrinous degeneration, stopping of caseous necrosis, and improvement in alveolar spaces. The mentioned results, according to the authors, suggested a pro-healing impact of inhaled 1,25(OH)2-VD3 on lung tissue damage caused by *M. tuberculosis* [50]. It is also worth mentioning the data collected by Serre et al. during an investigation of the influence of a single dose of 1,25(OH)2-VD3 (200 pg/g) administered directly to the respiratory tract by aerosolizer syringe on LPS-induced acute lung inflammation in male C57BL/6JolaH mice on standard and VD3-deficient diets. The mentioned studies revealed that in VD3-sufficient mice, 1,25(OH)2-VD3 reduced the acute inflammation caused by LPS, especially the total number of immune cells in BAL fluid, and decreased the elevated by LPS-level of CXCL5. On the contrary, in severe VD3-deficient mice, 1,25(OH)2-VD3 was unable to inhibit infiltration of immune cells into the respiratory tract but effectively prevented epithelial barrier leakage and lung damage. Similar to our study, Serre et al. investigated the concentration of several cytokines, including IL1β, IL6, IL10, IL13, IFNγ, IL17A, and TNFα. Nevertheless, the only statistically significant differences were observed in the concentration of IL13 in BAL fluid and lung homogenates collected from VD3-deficient mice, and a significant decrease of these anti-inflammatory cytokines in response to 1,25(OH)2-VD3 intervention, which may explain the mentioned failure in attenuation of inflammatory cell infiltration into the lungs [51].

**Figure 8:**
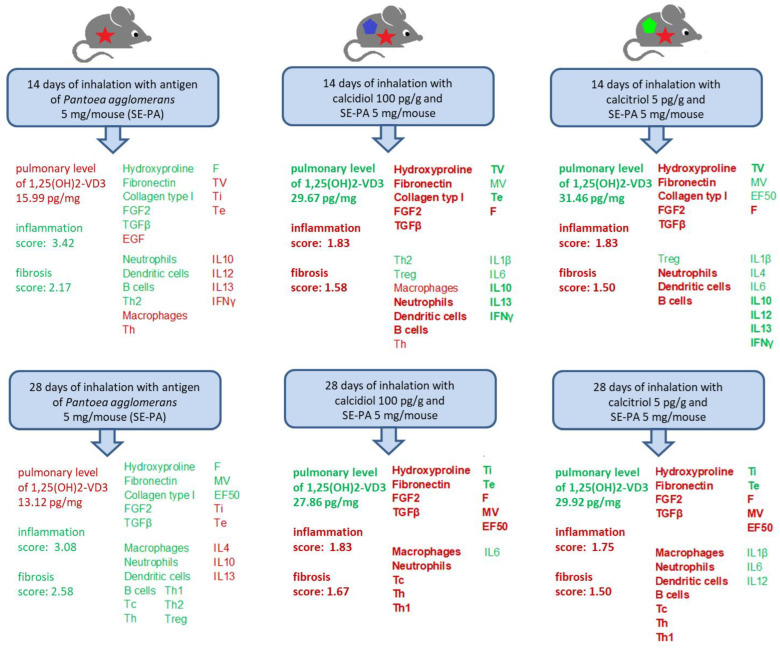
Anti-inflammatory and antifibrotic effect of inhaled 25(OH)-VD3 and 1,25(OH)2-VD3 in the murine model of HP. Parameters that increased in response to the analyzed factors (VD3-deficiency, antigen of *P. agglomerans*, 25(OH)-VD3, 1,25(OH)2-VD3) are marked in green, while those that decreased are marked in red. Changes induced by vitamin D3 metabolites’ treatment that were crucial for inhibition of HP development are shown in bold. Red star symbolizes SE-PA inhalation. Blue and green pentagons symbolize inhalation with 25(OH)-VD3 and 1,25(OH)2-VD3, respectively.

A graphical summary of the most important beneficial changes, in the respiratory system of VD3-deficient mice with induced HP, caused by chronic exposure to 25(OH)-VD3 and 1,25(OH)2-VD3 was presented in the Figure 8. 

Although the results of our research encourage the use of vitamin D3 metabolites administered by inhalation in the treatment of HP, it should be emphasized that the proposed therapeutic strategy is based on the use of metabolites in concentrations enabling the restoration of the physiological concentration of 1,25(OH)2-VD3 in the lungs. The doses of 25(OH)-VD3 and 1,25(OH)2-VD3 used in the study were selected experimentally and based on the above-mentioned criteria [35]. Consequently, the implementation of therapy based on inhalation of vitamin D3 metabolites will also require, in addition to determining the concentration of 1,25(OH)2-VD3 in the lungs, the development of models allowing the selection of a therapeutic dose in connection with the detected 1,25(OH)2-VD3 level. In addition, it will also be necessary to implement techniques for monitoring 1,25(OH)2-VD3 in the respiratory tract, or to develop models to establish the correlation of the pulmonary 1,25(OH)2-VD3 level with its serum concentration. Another limitation of the presented study is that it was performed only on male mice. Recent studies indicated gender as an important factor influencing VD3 metabolism, and consequently, its biological activity [55]. Men have shown lower levels of 7-dehydrocholesterol and vitamin D3 transporting protein (DBP), as well as higher expression of the enzyme CYP24A1 responsible for catabolism of 25(OH)-VD3 and 1,25(OH)2-VD3. On the contrary, women have shown higher levels of the vitamin D3 receptor and enzyme CYP27B1, responsible for the conversion of 25(OH)-VD3 to the biologically active form 1,25(OH)2-VD3. The indicated changes are associated with the sex hormones, among which testosterone inhibits vitamin D metabolism, while estrogens stimulate this process [55]. In the light of these data, additional studies on female mice are recommended to verify the obtained results. Finally, it is worth mentioning another important and quite obvious limitation of the presented research, which is the animal model of HP. Despite the fact that humans and mice share many similarities in their anatomy and physiology, there are also some differences which could be the key in the light of the presented study. Humans are able to abundantly synthesize vitamin D3 in their skin, while mice, as a nocturnal species covered with fur, obtain the majority of vitamin D from food. Moreover, there are significant differences in the composition, expression, and regulation of the VDR between humans and mice, which impact the biological features of vitamin D3. There is only one murine VDR protein, and only the liganded VDR autoregulates the expression of the *Vdr* gene and level of VDR protein. In humans, this system is much more complicated, there are several isoforms of VDR and several nuclear receptors, and their ligands regulate the expression of the *VDR* gene, and thus, the amount of VDR proteins [56]. Consequently, the final confirmation of the obtained results, like any other therapy, will require human trials.

## 4. Materials and Methods

### 4.1. Reagents

Unless otherwise indicated, the chemicals used in the study were purchased from Sigma-Aldrich Co. LLC. Detailed description of the preparation of *Pantoea agglomerans* extract has been presented previously [25,31]. In short, *P. agglomerans* strain M-10-3 was inoculated on enriched nutrient agar medium (BTL, Łódź, Poland) supplemented with peptides (Proteobak, BTL, Łódź, Poland) and then incubated for 72 h at 37 °C. The bacterial mass was harvested, homogenized, and extracted in saline in the proportion 1:2 for 48 h at 4 °C, with intermittent disruption of cells by freezing and thawing procedures. Afterward, the supernatant was separated by centrifugation at 10,000 rpm at 4 °C, and finally lyophilized. The study was conducted using saline extract of the *P. agglomerans* (SE-PA), obtained by dissolving 30 mg of *P. agglomerans* cells lyophilizate in 10 mL of PBS just before use. SE-PA contains proteins, sugars, DNA, and RNA (42.3%, 15.2%, 0.018%, and 0.014%, respectively, as determined by spectrophotometric analysis) and a relatively small quantity of active endotoxin (1% as assessed by Limulus test (Pyroquant Diagnostik GmbH, Germany) [33].

### 4.2. Design of Study

Three-month-old male C57BL/6 mice were purchased from the Mossakowski Medical Research Centre of the Polish Academy of Sciences in Warsaw, Poland. The mice were kept in colony cages with access to food and tap water ad libitum, under standardized housing conditions (natural light-dark cycle, a temperature of 22–24 °C). The mice belonging to the main control were on a diet with standard cholecalciferol level (0.5 IU/g), while mice belonging to the other research groups were given a VD3-deficient diet with a reduced cholecalciferol level (0.05 IU/g). Animal feed purchased from Altromin (Altromin Spezialfutter GmbH & Co. KG, Lage, Germany) was standardized and strictly controlled for vitamin D presence. Experiments were preceded by a one week of acclimatization (getting used to the new environment and the smell and touch of researchers), followed by an additional week’s period during which animals were tamed to inhalation chambers and accessories (neck restrainers and nose–mouth silicon masks) by gradually extending the time spent in chambers with dedicated equipment. After the acclimatization and adaptation procedures, the actual experiment began. The mice were exposed to a saline extract of *Pantoea agglomerans* at doses of 5 mg/mouse (SE-PA), 25(OH)-VD3 at a concentration of 100 pg/g or 1,25(OH)2-VD3 at a concentration of 5 pg/g. SE-PA and vitamin D3 metabolites were administered in 10 mL phosphate-buffered saline via nebulization for 14 or 28 consecutive days using the Buxco Inhalation Tower (Data Sciences International, St. Paul, MN, USA) under the following conditions: airflow 2.5 L/min; pressure −0.5 cm H_2_O; room temperature; average nebulization rate 353 µL/min. Because of specific features of the used research model in which strong inflammation typical for acute HP is observed between 7 and 14 days of inhalation, while signs of fibrosis characteristic for chronic HP are noted from the 28th day of inhalation, the data were collected after the above-indicated 14 and 28 days of treatment. At the end of each experiment, the mice were placed in a Non-invasive Airway Mechanics Plethysmograph Chamber with Allay Restraint and Halcon Technology (Data Sciences International, St. Paul, MN, USA). Pulmonary function of the mice was monitored under the following conditions: airflow 0.6 l/min; pressure 0 cm H_2_O; room temperature. Data were collected every 2 s for 10 min and analyzed. The frequency of breathing (F), tidal volume (TV), minute volume (MV), inspiratory time (Ti), expiratory time (Te), and mid-tidal expiratory flow (EF50) were determined using DSI FinePointe Software v 2.3.1.21. Each experiment ended with the humane killing of the mice via cervical dislocation with spinal cord rupture, followed by dissection and the collection of samples of blood and lung for further examination. The research group description is presented in Table 1. Experimental protocols were approved by the Local Ethics Committee for Animal Experimentation in Lublin, Poland (Resolution Nos. 28/2021, 25/2022, and 73/2022).

### 4.3. Measurement of 1,25(OH)2-VD3 Concentration—ELISA Method

After clotting, the blood samples were centrifugated (25,000× *g*, 10 min, 20 °C) to obtain the serum. 1,25(OH)2-VD3 concentration in serum was examined by the Mouse 1,25-dihydroxyvitamin D3(DVD/DHVD3) ELISA following manufacturer instruction (Shanghai Coon Koon Biotech Co., Ltd., Shanghai, China).

Lung samples were placed in Lysing Matrix M tubes (MP Biomedicals, Solon, OH, USA) containing 5 mM EDTA solution in PBS (0.5 mL/tube). Tissues were then homogenized mechanically using a FastPrep-24 5G homogenizer (MP Biomedicals) under the following conditions: 6 m/s, 40 s, and 20 °C. The homogenates were further incubated on ice for 20 min and centrifugated (10,000× *g*, 5 min, 4 °C). The obtained supernatants were next passed through a 70 µm nylon mesh into new tubes. Determination of 1,25(OH)2-Vd3 concentration in lung tissue homogenates was preceded by its extraction using the Extraction Kit for 1,25(OH)2 Vitamin D ELISA, after which proper investigation using 1,25(OH)2 Vitamin D Total ELISA according to the manufacturer’s instructions (BioVendor R&D, Brno, Czech Republic). Because of the significant differences in the weight of the lungs used in the assays, obtained results were normalized to the total protein content in lung homogenates determined using the standard BCA Protein Assay Kit (Pierce Biotechnology, Rockford, IL, USA).

### 4.4. Determination of Subpopulations of Pulmonary Immune Cells—Flow Cytometry

Murine lung tissue obtained during dissection were cut manually into small pieces and placed in RPMI medium supplemented with 0.5 mg/mL of collagenase type IV, 0.025 mg/mL of elastase and 25 U/mL of DNase I (Roche Diagnostics, Mannheim, Germany). Enzymatic digestion of lung tissue was carried out on a rotor for one hour at RT. RPMI medium was then supplemented with 10% of Fetal Bovine Serum (FBS), added to stop lysis. Obtained lung tissue homogenates were passed through a 70-μm filter and centrifugated (500 × G; 10 min 20 °C). Erythrocytes presented in the obtained cell mixture were removed using the Human Erythrocyte Lysing Kit, following the manufacturer’s instructions (R&D Systems, Inc., Abingdon, UK). The obtained cell mixture was resuspended in a cytometer buffer (PBS with 2% of FBS) and subjected to cytometric analysis.

The following markers were examined to analyze the phenotype of isolated lung cells: lymphocytes Th1 (CD3, CD4, IFNγ), lymphocytes Th2 (CD3, CD4, IL4), lymphocytes Tc (CD3, CD8), lymphocytes Treg (CD3, CD4, CD25, FoxP3), lymphocytes B (B220, CD19), macrophages M1 (CD11b, F4/80, CD86), macrophages M2 (CD11b, F4/80, CD206) neutrophils (CD11b, Ly-6G/Gr-1), and dendritic cells (CD103, CD11b, CD209). Antibodies and their isotype controls were obtained from BD Biosciences (San Diego, CA, USA), except antibodies against CD206 and corresponding isotype controls, which were purchased from Life Technologies Corporation (Carlsbad, CA, USA). In the case of surface antigen staining, cells were incubated with specific monoclonal antibodies for 20 min in the dark at RT. To determine lymphocytes Treg, after staining of surface antigens (CD3, CD4, and CD25), cells were subjected to a permeabilization procedure using the Mouse Regulatory T Cell Staining Kit (eBiosciences, San Diego, CA, USA), according to the manufacturer’s instructions. The cells were then stained with antibodies specific for FoxP3 (incubation in the dark for 20 min at RT). In the case of lymphocytes Th1 and Th2 phenotyping, the mixture of pulmonary cells was suspended in RPMI medium supplemented with 10% FBS, phorbol-12-myristate-13-acetate, and ionomycin, and incubated for 4 h at 37 °C. After the first hour of incubation, the cell culture medium was additionally supplemented with monensin (BD Biosciences, San Jose, CA, USA). Next, the cells were incubated with anti-CD4 monoclonal antibody for 20 min at RT. After permeabilizing the cells using reagents from the Fixation/Permeabilization Kit (BD Biosciences, San Jose, CA, USA), cells were stained with antibodies specific for IFNγ and IL4 (incubation in the dark for 30 min at RT). Regardless of the type of staining, unbounded antibodies were removed by cell suspension with PBS and centrifugation (500× *g*; 10 min 20°). In the final steps, stained cells were suspended in a flow cytometry buffer and analyzed using flow cytometer BD Accuri C6 Plus (BD Biosciences, San Jose, CA, USA). A minimum of 1 × 10^5^ cells was acquired and analyzed by BD CSampler Plus software v 1.0.34.1 (BD Biosciences, San Jose, CA, USA). As a negative control, the unstained cells of each sample were used. The gates for individual markers were inserted based on the isotype control signal to exclude background staining. Specific isotype controls were used for each tested marker. The gating strategies are presented in the Supplementary Materials: Figure S1 (surface staining) and Figure S2 (intracellular staining).

### 4.5. Examination of Signs of Inflammation and Fibrosis in Lung Tissue—Hematoxylin and Eosin Staining and Masson Trichrome Staining

Lung tissue samples were fixed and stored in 4% buffered formalin. After dehydration, lung samples were embedded in paraffin wax. Next, 5 μm-thick sections were obtained from the paraffin blocks and stained with hematoxylin and eosin (H&E) or Masson trichrome. Microscopic preparations were evaluated by light microscopy to assess their general morphology and range of inflammation (H&E staining) or to determine signs of fibrosis (Masson Trichrome staining). Histological examination was performed by a pathologist who was blinded to the experimental protocol. Lung injury was scored according to features of inflammation and fibrosis. These features were graded with the five-point Murray scale: 0 = regular tissue; 1 = slight injury 25%; 2 = moderate injury 50%; 3 = severe injury 75%; 4 = very severe injury 100%. The mentioned parameters were analyzed twice and the final scores were calculated as the mean of the investigated items in each research group. Lung tissue images were captured with the light microscope MW 50 (OPTA-TECH, Warsaw, Poland), while micrographs were prepared in Capture V2.0 software (OPTA-TECH, Warsaw, Poland).

### 4.6. Measurement of Selected Protein Concentration—ELISA Method

The detailed description of the preparation of lung tissue homogenates has been presented previously [25]. The concentration of proteins in the obtained homogenates was measured by the ELISA kits designed to detect: Mouse Cathelicidin Antimicrobial Peptide (CAMP); Mouse Collagen Type I (COL1); Mouse Epidermal Growth Factor (EGF); Mouse basic Fibroblast Growth Factor (FGF2); Hydroxyproline (Hyp); Mouse Fibronectin (FN); Mouse Transforming Growth Factor Beta 1 (TGFb1); Mouse Interferon Gamma (IFNg); Mouse Interleukin 1 Beta (IL1b); Mouse Interleukin 4 (IL4); Mouse Interleukin 6 (IL6); Mouse Interleukin 10 (IL10); Mouse Interleukin 12A (IL12A); Mouse Interleukin 13 (IL13). All ELISA kits were purchased from Cloud-Clone Corp., Katy, TX, USA) according to the manufacturer’s instructions.

### 4.7. Statistical Analysis

Statistical analyses were performed using Microsoft Excel 2019 and GraphPad Prism 5.0. Data were analyzed with One-way ANOVA followed by multiple-group comparison analysis with a two-tailed unpaired *t*-test or in the case of flow cytometry data the Mann–Whitney U-test. Moreover, the column statistics were used for comparisons. The vertical segments in box plots show the first quartile, median, and third quartile. The whiskers on both ends represent the maximum and minimum values for each data set analyzed. A *p* value < 0.05 was considered statistically significant.

## 5. Conclusions

In summary, the performed study revealed that inhalations with 25(OH)-VD3 and 1,25(OH)2-VD3 effectively eliminated most of the observed negative changes in the respiratory system caused by vitamin D3 deficiency, and these beneficial features were associated with their ability to restore the physiological concentration of 1,25(OH)2-VD3 in the mice lung tissue. First of all, 25(OH)VD3 and 1,25(OH)2-VD3 treatment in VD3-deficient mice also exposed to *P. agglomerans* antigen demonstrated that tested vitamin D3 metabolites eliminated, or at least weakened, the negative effects of HP causative factor, and thus, inhibited the disease development. The beneficial influence of HP therapy based on inhalation of vitamin D3 metabolites, in doses restoring the physiological level of 1,25(OH)2-VD3 in the pulmonary compartments, included improvement of respiratory functions and attenuation of inflammation and signs of fibrosis. Moreover, the presented study is the first one showing the influence of vitamin D3 deficiency on HP development, including stimulation of inflammation and fibrosis.

Despite the discovery of beneficial features of inhalations with vitamin D3 metabolites on respiratory response in the murine model of HP, obtained data require verification in the clinical study. Thus, the presented results should be treated as the first step in the development of a new, effective, and safe therapy for HP.

## Figures and Tables

**Table 1:** Description of research groups.

Research Group (Number of Animals)	Vitamin D3 Content in Diet	Calcidiol; 25(OH)-Vitamin D3 (100 pg/g)	Calcitriol; 1,25(OH)_2_-Vitamin D3 (5 pg/g)	SE-PA; Saline Extract of *Pantoea agglomerans* (5 mg/Mouse)
Main control 0 days (*n* = 6)	0.5 IU/g	-	-	-
Control 0 days (*n* = 6)	0.05 IU/g	-	-	-
SE-PA 14 days (*n* = 6)	0.05 IU/g	-	-	30 min daily inhalation for 14 consecutive days
SE-PA 28 days (*n* = 6)	0.05 IU/g	-	-	30 min daily inhalation for 28 consecutive days
25(OH)-VD3 14 days (*n* = 6)	0.05 IU/g	30 min daily inhalation for 14 consecutive days	-	-
25(OH)-VD3 28 days (*n* = 6)	0.05 IU/g	30 min daily inhalation for 28 consecutive days	-	-
1,25(OH)2-VD3 14 days (*n* = 6)	0.05 IU/g	-	30 min daily inhalation for 14 consecutive days	-
1,25(OH)2-VD3 28 days (*n* = 5)	0.05 IU/g	-	30 min daily inhalation for 28 consecutive days	-
SE-PA+ 25(OH)-VD3 14 days (*n* = 6)	0.05 IU/g	30 min daily inhalation for 14 consecutive days	-	30 min daily inhalation for 14 consecutive days
SE-PA+ 25(OH)-VD3 28 days (*n* = 6)	0.05 IU/g	30 min daily inhalation for 28 consecutive days	-	30 min daily inhalation for 28 consecutive days
SE-PA+ 1,25(OH)2-VD3 14 days (*n* = 6)	0.05 IU/g	-	30 min daily inhalation for 14 consecutive days	30 min daily inhalation for 14 consecutive days
SE-PA+ 1,25(OH)2-VD3 28 days (*n* = 6)	0.05 IU/g	-	30 min daily inhalation for 28 consecutive days	30 min daily inhalation for 28 consecutive days

## Data Availability

All data analyzed during this study are included in this article. Further inquiries can be directed to the corresponding author.

## Supplementary Materials

The following supporting information can be downloaded at: https://www.mdpi.com/article/10.3390/ijms251910289/s1.

## References

[1] Holick M.F. (2017). The Vitamin D deficiency pandemic: Approaches for diagnosis, treatment and prevention. Rev. Endocr. Metab. Disord..

[2] Christakos S., Dhawan P., Verstuyf A., Verlinden L., Carmeliet G. (2016). Vitamin D: Metabolism, molecular mechanism of action, and pleiotropic effects. Physiol. Rev..

[3] Norman A.W. (2008). From vitamin D to hormone D: Fundamentals of the vitamin D endocrine system essential for good health. Am. J. Clin. Nutr..

[4] Ebeling P.R., Adler R.A., Jones G., Liberman U.A., Mazziotti G., Minisola S., Munns C.F., Napoli N., Pittas A.G., Giustina A. (2018). Management of endocrine disease: Therapeutics of Vitamin D. Eur. J. Endocrinol..

[5] Theodoratou E., Tzoulaki I., Zgaga L., Ioannidis J.P. (2014). Vitamin D and multiple health outcomes: Umbrella review of systematic reviews and meta-analyses of observational studies and randomised trials. BMJ.

[6] Holick M.F., Chen T.C. (2008). Vitamin D deficiency: A worldwide problem with health consequences. Am. J. Clin. Nutr..

[7] Chang S.W., Lee H.C. (2019). Vitamin D and health—The missing vitamin in humans. Pediatr. Neonatol..

[8] Chojnacki M., Lemieszek M.K. (2023). Role of vitamin D_3_ in selected pulmonary diseases with particular emphasis on lung fibrosis. Ann. Agric. Environ. Med..

[9] Entrenas-Castillo M., Salinero-González L., Entrenas-Costa L.M., Andújar-Espinosa R. (2022). Calcifediol for use in treatment of respiratory disease. Nutrients.

[10] Ganmaa D., Enkhmaa D., Nasantogtokh E., Sukhbaatar S., Tumur-Ochir K.E., Manson J.E. (2022). Vitamin D, respiratory infections, and chronic disease: Review of meta-analyses and randomized clinical trials. J. Intern. Med..

[11] Ahmad S., Arora S., Khan S., Mohsin M., Mohan A., Manda K., Syed M.A. (2021). Vitamin D and its therapeutic relevance in pulmonary diseases. J. Nutr. Biochem..

[12] Luong K.V., Nguyen L.T. (2013). Beneficial role of vitamin D_3_ in the prevention of certain respiratory diseases. Ther. Adv. Respir. Dis..

[13] Costabel U., Miyazaki Y., Pardo A., Koschel D., Bonella F., Spagnolo P., Guzman J., Ryerson C.J., Selman M. (2020). Hypersensitivity pneumonitis. Nat. Rev. Dis. Primers.

[14] Morell F., Ojanguren I., Cruz M.J. (2019). Diagnosis of occupational hypersensitivity pneumonitis. Curr. Opin. Allergy Clin. Immunol..

[15] Greenberger P.A. (2019). Hypersensitivity pneumonitis: A fibrosing alveolitis produced by inhalation of diverse antigens. J. Allergy Clin. Immunol..

[16] Calaras D., David A., Vasarmidi E., Antoniou K., Corlateanu A. (2024). Hypersensitivity Pneumonitis: Challenges of a Complex Disease. Can. Respir. J..

[17] Cottin V., Hirani N.A., Hotchkin D.L., Nambiar A.M., Ogura T., Otaola M., Skowasch D., Park J.S., Poonyagariyagorn H.K., Wuyts W. (2018). Presentation, diagnosis and clinical course of the spectrum of progressive-fibrosing interstitial lung diseases. Eur. Respir. Rev..

[18] Salisbury M.L., Myers J.L., Belloli E.A., Kazerooni E.A., Martinez F.J., Flaherty K.R. (2017). Diagnosis and Treatment of Fibrotic Hyper-sensitivity Pneumonia. Where We Stand and Where We Need to Go. Am. J. Respir. Crit. Care Med..

[19] Vasakova M., Morell F., Walsh S., Leslie K., Raghu G. (2017). Hypersensitivity Pneumonitis: Perspectives in Diagnosis and Management. Am. J. Respir. Crit. Care Med..

[20] Ghaseminejad-Raeini A., Ghaderi A., Sharafi A., Nematollahi-Sani B., Moossavi M., Derakhshani A., Sarab G.A. (2023). Immunomodulatory actions of vitamin D in various immune-related disorders: A comprehensive review. Front. Immunol..

[21] Ao T., Kikuta J., Ishii M. (2021). The effects of vitamin D on immune system and inflammatory diseases. Biomolecules.

[22] Charoenngam N., Holick M.F. (2020). Immunologic effects of vitamin D on human health and disease. Nutrients.

[23] Golec M., Lemieszek M.K., Dutkiewicz J., Milanowski J., Barteit S. (2022). A scoping analysis of cathelicidin in response to organic dust exposure and related chronic lung illnesses. Int. J. Mol. Sci..

[24] Lemieszek M.K., Golec M., Zwoliński J., Dutkiewicz J., Milanowski J. (2022). Cathelicidin treatment silences epithelial-mesenchymal transition involved in pulmonary fibrosis in a murine model of hypersensitivity pneumonitis. Int. J. Mol. Sci..

[25] Lemieszek M.K., Sawa-Wejksza K., Golec M., Dutkiewicz J., Zwoliński J., Milanowski J. (2021). Beneficial impact of cathelicidin on hypersensitivity pneumonitis treatment—In vivo studies. PLoS ONE.

[26] Zheng S., Yang J., Hu X., Li M., Wang Q., Dancer R.C.A., Parekh D., Gao-Smith F., Thickett D.R., Jin S. (2020). Vitamin D attenuates lung injury via stimulating epithelial repair, reducing epithelial cell apoptosis and inhibits TGF-β induced epithelial to mesenchymal transition. Biochem. Pharmacol..

[27] Fischer K.D., Agrawal D.K. (2014). Vitamin D regulating TGF-β induced epithelial-mesenchymal transition. Respir. Res..

[28] Upadhyay S.K., Verone A., Shoemaker S., Qin M., Liu S., Campbell M., Hershberger P.A. (2013). 1,25-dihydroxyvitamin D_3_ (1,25(OH)_2_D_3_) signaling capacity and the epithelial-mesenchymal transition in non-small cell lung cancer (NSCLC): Implications for use of 1,25(OH)_2_D_3_ in NSCLC treatment. Cancers.

[29] Ramirez A.M., Wongtrakool C., Welch T., Steinmeyer A., Zügel U., Roman J. (2010). Vitamin D inhibition of pro-fibrotic effects of transforming growth factor beta1 in lung fibroblasts and epithelial cells. J. Steroid. Biochem. Mol. Biol..

[30] Lemieszek M.K., Rzeski W., Golec M., Mackiewicz B., Zwoliński J., Dutkiewicz J., Milanowski J. (2020). *Pantoea agglomerans* chronic exposure induces epithelial-mesenchymal transition in human lung epithelial cells and mice lungs. Ecotoxicol. Environ. Saf..

[31] Lemieszek M.K., Chilosi M., Golec M., Skórska C., Dinnyes A., Mashayekhi K., Vierlinger K., Huaux F., Wielscher M., Hofner M. (2013). Age influence on hypersensitivity pneumonitis induced in mice by exposure to *Pantoea agglomerans*. Inhal. Toxicol..

[32] Lemieszek M., Chilosi M., Golec M., Skórska C., Huaux F., Yakoub Y., Pastena C., Daniele I., Cholewa G., Sitkowska J. (2011). Mouse model of hypersensitivity pneumonitis after inhalation exposure to different microbial antigens associated with organic dusts. Ann. Agric. Environ. Med..

[33] Dutkiewicz J., Mackiewicz B., Lemieszek M.K., Golec M., Skórska C., Góra-Florek A., Milanowski J. (2016). *Pantoea agglomerans*: A mysterious bacterium of evil and good. Part II--Deleterious effects: Dust-borne endotoxins and allergens--focus on grain dust, other agricultural dusts and wood dust. Ann. Agric. Environ. Med..

[34] Walkin L., Herrick S.E., Summers A., Brenchley P.E., Hoff C.M., Korstanje R., Margetts P.J. (2013). The role of mouse strain differences in the susceptibility to fibrosis: A systematic review. Fibrogenesis Tissue Repair.

[35] Chojnacki M., Anisiewicz J., Leśniowska I., Lemieszek M.K. (2023). Inhalation with vitamin D_3_ metabolites—A novel strategy to restore vitamin D_3_ deficiencies in lung tissue. Appl. Sci..

[36] Fleet J.C., Gliniak C., Zhang Z., Xue Y., Smith K.B., McCreedy R., Adedokun S.A. (2008). Serum metabolite profiles and target tissue gene expression define the effect of cholecalciferol intake on calcium metabolism in rats and mice. J. Nutr..

[37] Shi Y., Liu T., Yao L., Xing Y., Zhao X., Fu J., Xue X. (2017). Chronic vitamin D deficiency induces lung fibrosis through activation of the renin-angiotensin system. Sci. Rep..

[38] Zosky G.R., Berry L.J., Elliot J.G., James A.L., Gorman S., Hart P.H. (2011). Vitamin D deficiency causes deficits in lung function and alters lung structure. Am. J. Respir. Crit. Care. Med..

[39] Nuñez N.K., Bennett E., Chen L., Pitrez P.M., Zosky G.R. (2017). The independent effects of vitamin D deficiency and house dust mite exposure on lung function are sex-specific. Sci. Rep..

[40] Seldeen K.L., Pang M., Leiker M.M., Bard J.E., Rodríguez-Gonzalez M., Hernandez M., Sheridan Z., Nowak N., Troen B.R. (2018). Chronic vitamin D insufficiency impairs physical performance in C57BL/6J mice. Aging.

[41] Holick M.F. (2020). Sunlight, UV radiation, vitamin D, and skin cancer: How much sunlight do we need?. Adv. Exp. Med. Biol..

[42] Young A.R., Claveau J., Rossi A.B. (2017). Ultraviolet radiation and the skin: Photobiology and sunscreen photoprotection. J. Am. Acad. Dermatol..

[43] Holick M.F., Binkley N.C., Bischoff-Ferrari H.A., Gordon C.M., Hanley D.A., Heaney R.P., Murad M.H., Weaver C.M., Endocrine Society (2011). Evaluation, treatment, and prevention of vitamin D deficiency: An Endocrine Society clinical practice guideline. J. Clin. Endocrinol. Metab..

[44] Santos H.O., Martins C.E.C., Forbes S.C., Delpino F.M. (2023). A scoping review of vitamin D for nonskeletal health: A framework for evidence-based clinical practice. Clin. Ther..

[45] Sassi F., Tamone C., D’Amelio P. (2018). Vitamin D: Nutrient, hormone, and immunomodulator. Nutrients.

[46] Carlberg C. (2022). Vitamin D and its target genes. Nutrients.

[47] Slominski R.M., Stefan J., Athar M., Holick M.F., Jetten A.M., Raman C., Slominski A.T. (2020). COVID-19 and vitamin D: A lesson from the skin. Exp. Dermatol..

[48] Wang J., Slominski A., Tuckey R.C., Janjetovic Z., Kulkarni A., Chen J., Postlethwaite A.E., Miller D., Li W. (2012). 20-hydroxyvitamin D_3_ inhibits proliferation of cancer cells with high efficacy while being non-toxic. Anticancer Res..

[49] Slominski A.T., Janjetovic Z., Fuller B.E., Zmijewski M.A., Tuckey R.C., Nguyen M.N., Sweatman T., Li W., Zjawiony J., Miller D. (2010). Products of vitamin D_3_ or 7-dehydrocholesterol metabolism by cytochrome P450scc show anti-leukemia effects, having low or absent calcemic activity. PLoS ONE.

[50] Reddy D.V.S., Shafi H., Bharti R., Roy T., Verma S., Raman S.K., Verma K., Azmi L., Ray L., Singh J. (2022). Preparation and evaluation of low-dose 1,25(OH)_2_-VD_3_ dry powder inhalation as host-directed adjunct therapy for tuberculosis. Pharm. Res..

[51] Serré J., Mathyssen C., Ajime T.T., Heigl T., Verlinden L., Maes K., Verstuyf A., Cataldo D., Vanoirbeek J., Vanaudenaerde B. (2022). Local nebulization of 1α,25(OH)_2_D_3_ attenuates LPS-induced acute lung inflammation. Respir. Res..

[52] Akita T., Hirokawa M., Yamashita C. (2020). The effects of 1α,25-dihydroxyvitamin D_3_ on alveolar repair and bone mass in adiponectin-deficient mice. J. Steroid. Biochem. Mol. Biol..

[53] Horiguchi M., Hirokawa M., Abe K., Kumagai H., Yamashita C. (2016). Pulmonary administration of 1,25-dihydroxyvitamin D3 to the lungs induces alveolar regeneration in a mouse model of chronic obstructive pulmonary disease. J. Control Release.

[54] Taylor S.K., Sakurai R., Sakurai T., Rehan V.K. (2016). Inhaled vitamin D: A novel strategy to enhance neonatal lung maturation. Lung.

[55] Wierzbicka A., Oczkowicz M. (2022). Sex differeces in vitamin D metabolism, serum levels and action. Br. J. Nutr..

[56] Marcinkowska E. (2020). The vitamin D system in humans and mice: Similar but not the same. Reports.

