# Supplementary material for: Beneficial Impact of Inhaled 25(OH)-Vitamin D3 and 1,25(OH)2-Vitamin D3 on Pulmonary Response in the Murine Model of Hypersensitivity Pneumonitis
Source: Int J Mol Sci. 2024 Sep 24;25(19):10289. doi: 10.3390/ijms251910289 (PMC11476509; doi:10.3390/ijms251910289)
Supplement: Supplementary file 1 [file ijms-25-10289-s001.zip › Table S1.pdf]

Table S1. Changes in the pulmonary and serum levels of 1,25(OH)2-VD3 concentration in response to inhalation with antigen of *Pantoea agglomerans* and/or vitamin D3 metabolites. ELISA data are presented as a mean of calcitriol concentrations ± SD.

	**Main control 0 days**	**Control 0 days**	**SE-PA 14 days**	**SE-PA 28 days**	**25(OH)-VD3 14 days**	**25(OH)-VD3 28 days**	**1,25(OH)2-VD3 14 days**	**1,25(OH)2-VD3 28 days**	**SE-PA+ 25(OH)-VD3 14 days**	**SE-PA+ 25(OH)-VD3 28 days**	**SE-PA+ 1,25(OH)2-VD3 14 days**	**SE-PA+ 1,25(OH)2-VD3 28 days**
**pulmonary level of 1,25(OH)2-VD3 [pg/mg]**	30.31 ± 0.49	18.20 ± 0.81	15.99 ± 1.96	13.12 ± 2.68	30.29 ± 1.77	31.83 ± 3.06	31.08 ± 2.60	32.09 ± 2.71	29.67 ± 2.02	27.86 ± 2.72	31.46 ± 3.05	29.92 ± 2.62
**serum level of 1,25(OH)2-VD3 [pg/ml]**	132.2 ± 6.55	98.6 ± 9.48	73.35± 11.55	65.7 ± 12.23	129.2 ± 13.36	132.1 ± 15.39	124.0 ± 14.25	126.8 ± 7.86	127.7 ± 7.77	124.5 ± 4.84	130.3 ± 7.90	120.3 ± 4.69
