# Supplementary material for: Beneficial Impact of Inhaled 25(OH)-Vitamin D3 and 1,25(OH)2-Vitamin D3 on Pulmonary Response in the Murine Model of Hypersensitivity Pneumonitis
Source: Int J Mol Sci. 2024 Sep 24;25(19):10289. doi: 10.3390/ijms251910289 (PMC11476509; doi:10.3390/ijms251910289)
Supplement: Supplementary file 1 [file ijms-25-10289-s001.zip › Table S2.pdf]

Table S2. Changes in the respiratory parameter in response to inhalation with antigen of *Pantoea agglomerans* and/or vitamin D3 metabolites. Whole-body plethysmography data are presented as a mean of respiratory parameter ± SD. F- frequency of breathing, TV-tidal volume, MV-minute volume, Ti-time of inspiratory, Te-time of expiratory, EF50-mid-tidal expiratory flow

	**Main control 0 days**	**Control 0 days**	**SE-PA 14 days**	**SE-PA 28 days**	**25(OH)-VD3 14 days**	**25(OH)-VD3 28 days**	**1,25(OH)2-VD3 14 days**	**1,25(OH)2-VD3 28 days**	**SE-PA+ 25(OH)-VD3 14 days**	**SE-PA+ 25(OH)-VD3 28 days**	**SE-PA+ 1,25(OH)2-VD3 14 days**	**SE-PA+ 1,25(OH)2-VD3 28 days**
**F [breaths/min]**	266.72 ± 22.97	291.40 ± 10.79	361.13 ± 39.40	414.28 ± 33.62	296.47 ± 22.63	283.97 ± 21.88	289.53 ± 15.28	266.85 ± 15.07	304.52 ± 27.99	294.93 ± 42.53	337.12 ± 18.62	323.60 ± 34.51
**TV [ml]**	0.310 ± 0.061	0.330 ± 0.027	0.206 ± 0.035	0.328 ± 0.071	0.366 ± 0.039	0.350 ± 0.032	0.364 ± 0.024	0.316 ± 0.041	0.286 ± 0.043	0.299 ± 0.010	0.342 ± 0.027	0.336 ± 0.024
**MV [ml/min]**	82.94 ± 21.27	95.14 ± 7.44	71.50 ± 6.01	133.84 ± 20.63	109.27 ± 13.87	100.75 ± 16.50	106.19 ± 10.77	85.02 ± 15.43	88.32 ± 16.50	89.96 ± 14.74	116.72 ± 12.97	109.99 ± 14.87
**Ti [s]**	0.104 ± 0.013	0.090 ± 0.004	0.072 ± 0.011	0.069 ± 0.004	0.091 ± 0.010	0.094 ± 0.007	0.092 ± 0.007	0.101 ± 0.012	0.079 ± 0.005	0.083 ± 0.006	0.074 ± 0.004	0.079 ± 0.006
**Te [s]**	0.138 ± 0.015	0.129 ± 0.006	0.112 ± 0.008	0.079 ± 0.008	0.123 ± 0.011	0.136 ± 0.012	0.140 ± 0.013	0.138 ± 0.008	0.137 ± 0.022	0.151 ± 0.037	0.119 ± 0.021	0.123 ± 0.020
**EF50 [ml/s]**	3.536 ± 0.880	3.990 ± 0.245	3.590 ± 0.172	5.886 ± 0.618	4.375 ± 0.496	4.251 ± 0.570	4.621 ± 0.388	3.656 ± 0.666	3.631 ± 0.622	3.930 ± 0.527	4.759 ± 0.416	4.569 ± 0.674
