# Supplementary material for: Beneficial Impact of Inhaled 25(OH)-Vitamin D3 and 1,25(OH)2-Vitamin D3 on Pulmonary Response in the Murine Model of Hypersensitivity Pneumonitis
Source: Int J Mol Sci. 2024 Sep 24;25(19):10289. doi: 10.3390/ijms251910289 (PMC11476509; doi:10.3390/ijms251910289)
Supplement: Supplementary file 1 [file ijms-25-10289-s001.zip › Table S3.pdf]

Table S3. Quantification of inflammation and fibrosis in murine lung tissue in response to inhalation with antigen of *Pantoea agglomerans* and/or vitamin D3 metabolites. Data for histologic scores are given as mean ± SD of investigated items. These features were graded with 5-point Murray's scale: 0 = regular tissue; 1 = slight injury 25%; 2 = moderate injury 50%; 3 = severe injury 75%; 4 = very severe injury 100%.

	Main control 0 days	Control 0 days	SE-PA 14 days	SE-PA 28 days	25(OH)-VD3 14 days	25(OH)-VD3 28 days	1,25(OH)2-VD3 14 days	1,25(OH)2-VD3 28 days	SE-PA+ 25(OH)-VD3 14 days	SE-PA+ 25(OH)-VD3 28 days	SE-PA+ 1,25(OH)2-VD3 14 days	SE-PA+ 1,25(OH)2-VD3 28 days
**Inflammation score**	0.00 ± 0.00	0.00 ± 0.00	3.42 ± 0.51	3.08 ± 0.29	0.00 ± 0.00	0.25 ± 0.45	0.00 ± 0.00	0.10 ± 0.32	1.83 ± 0.39	1.83 ± 0.39	1.83 ± 0.39	1.75 ± 0.45
**Fibrosis score**	0.67 ± 0.49	1.00 ± 0.60	2.17 ± 0.39	2.58 ± 0.51	0.58 ± 0.51	0.83 ± 0.39	0.92 ± 0.67	0.90 ± 0.57	1.58 ± 0.51	1.67 ± 0.49	1.50 ± 0.52	1.50 ± 0.52
