# Supplementary material for: Beneficial Impact of Inhaled 25(OH)-Vitamin D3 and 1,25(OH)2-Vitamin D3 on Pulmonary Response in the Murine Model of Hypersensitivity Pneumonitis
Source: Int J Mol Sci. 2024 Sep 24;25(19):10289. doi: 10.3390/ijms251910289 (PMC11476509; doi:10.3390/ijms251910289)
Supplement: Supplementary file 1 [file ijms-25-10289-s001.zip › Table S4.pdf]

Table S4. Changes in the immune cell composition cytokine concentrations in murine lung tissue in response to inhalation with antigen of *Pantoea agglomerans* and/or vitamin D3 metabolites. Flow cytometry data are presented as a median of percentage of all immune cells

	**Main control 0 days**	**Control 0 days**	**SE-PA 14 days**	**SE-PA 28 days**	**25(OH)-VD3 14 days**	**25(OH)-VD3 28 days**	**1,25(OH)2-VD3 14 days**	**1,25(OH)2-VD3 28 days**	**SE-PA+ 25(OH)-VD3 14 days**	**SE-PA+ 25(OH)-VD3 28 days**	**SE-PA+ 1,25(OH)2-VD3 14 days**	**SE-PA+ 1,25(OH)2-VD3 28 days**
**Macrophages [%]**	18.80	7.190	14.55	27.47	12.86	8.94	10.30	9.26	12.56	11.12	14.68	14.30
**Neutrophils [%]**	6.27	3.71	16.37	34.47	4.47	4.34	6.13	5.90	10.53	8.68	9.08	5.19
**Dendritic cells [%]**	6.05	2.90	13.72	9.20	2.24	1.80	2.32	1.70	6.91	8.26	5.65	4.95
**Lymphocytes Tc [%]**	6.64	4.24	6.15	9.36	2.15	3.61	3.93	4.07	6.23	4.82	5.43	5.79
**Lymphocytes Th [%]**	6.89	2.88	5.12	11.23	3.52	2.28	2.70	2.43	3.85	5.72	4.41	5.57
**Lymphocytes B [%]**	0.36	0.19	1.01	0.87	0.05	0.44	0.30	0.35	0.50	0.84	0.36	0.49
**Lymphocytes Th1 [%]**	0.37	0.21	0.58	1.39	0.13	0.06	0.18	0.67	0.58	0.35	0.29	0.35
**Lymphocytes Th2 [%]**	0.06	0.08	0.46	0.57	0.05	0.03	0.06	0.69	0.81	0.15	0.25	0.21
**Lymphocytes Treg [%]**	0.02	0.02	0.31	0.46	0.17	0.00	0.05	0.04	0.25	0.70	0.72	0.60
