# Supplementary material for: Beneficial Impact of Inhaled 25(OH)-Vitamin D3 and 1,25(OH)2-Vitamin D3 on Pulmonary Response in the Murine Model of Hypersensitivity Pneumonitis
Source: Int J Mol Sci. 2024 Sep 24;25(19):10289. doi: 10.3390/ijms251910289 (PMC11476509; doi:10.3390/ijms251910289)
Supplement: Supplementary file 1 [file ijms-25-10289-s001.zip › Table S5.pdf]

Table S5. Changes in the concentration of components and modulators of extracellular matrix deposition in murine lung tissue in response to inhalation with antigen of *Pantoea agglomerans* and/or vitamin D3 metabolites. ELISA data are presented as a mean of protein concentrations ± SD.

	Main control 0 days	Control 0 days	SE-PA 14 days	SE-PA 28 days	25(OH)-VD3 14 days	25(OH)-VD3 28 days	1,25(OH)2-VD3 14 days	1,25(OH)2-VD3 28 days	SE-PA+ 25(OH)-VD3 14 days	SE-PA+ 25(OH)-VD3 28 days	SE-PA+ 1,25(OH)2-VD3 14 days	SE-PA+ 1,25(OH)2-VD3 28 days
**Hydroxyproline [ng/ml]**	3.72 ± 1.02	7.49 ± 1.26	15.97 ± 1.23	18.68 ± 1.49	6.84 ± 1.04	6.73 ± 1.30	5.19 ± 1.58	5.35 ± 0.86	11.13 ± 2.11	13.65 ± 1.53	10.87 ± 0.78	12.76 ± 1.11
**Fibronectin [ng/ml]**	58.17 ± 13.13	76.22 ± 5.13	82.67 ± 15.56	82.61 ± 16.63	49.03 ± 3.80	30.91 ± 4.35	44.26 ± 6.52	42.34 ± 17.34	60.54 ± 7.06	46.35 ± 8.53	60.32 ± 13.68	57.48 ± 6.24
**Collagen type I [pg/ml]**	1236.63 ± 127.93	1569.63 ± 134.44	1767.26 ± 261.03	1815.59 ± 205.70	1143.48 ± 258.20	1218.75 ± 212.81	762.27 ± 167.35	1348.79 ± 324.66	1109.22 ± 304.76	1642.31 ± 310.42	938.50 ± 154.00	1596.06 ± 243.69
**FGF2 [pg/ml]**	33.56 ± 8.09	79.70 ± 17.21	106.77 ± 13.49	138.27 ± 15.03	69.00 ± 10.44	67.88 ± 13.13	52.34 ± 15.95	53.89 ± 8.63	82.51 ± 12.60	103.16 ± 11.58	68.23 ± 7.88	77.17 ± 6.69
**EGF [pg/ml]**	296.02 ± 47.45	256.17 ± 43.38	100.94 ± 20.13	243.36 ± 54.13	142.22 ± 26.18	197.48 ± 30.96	111.80 ± 19.24	281.43 ± 27.68	121.88 ± 14.88	214.21 ± 38.26	99.62 ± 11.55	210.23 ± 37.45
**TGFβ [pg/ml]**	334.60 ± 41.95	400.47 ± 44.90	645.14 ± 63.66	672.21 ± 88.31	416.38 ± 54.16	421.66 ± 24.17	389.36 ± 53.59	421.44 ± 32.39	468.98 ± 61.75	535.25 ± 78.28	449.14 ± 52.52	526.66 ± 68.95
