# Supplementary material for: Beneficial Impact of Inhaled 25(OH)-Vitamin D3 and 1,25(OH)2-Vitamin D3 on Pulmonary Response in the Murine Model of Hypersensitivity Pneumonitis
Source: Int J Mol Sci. 2024 Sep 24;25(19):10289. doi: 10.3390/ijms251910289 (PMC11476509; doi:10.3390/ijms251910289)
Supplement: Supplementary file 1 [file ijms-25-10289-s001.zip › Table S6.pdf]

Table S6. Changes in the cytokine concentrations in murine lung tissue in response to inhalation with antigen of *Pantoea agglomerans* and/or vitamin D3 metabolites. ELISA data are presented as a mean of protein concentrations ± SD.

	**Main control 0 days**	**Control 0 days**	**SE-PA 14 days**	**SE-PA 28 days**	**25(OH)-VD3 14 days**	**25(OH)-VD3 28 days**	**1,25(OH)2-VD3 14 days**	**1,25(OH)2-VD3 28 days**	**SE-PA+ 25(OH)-VD3 14 days**	**SE-PA+ 25(OH)-VD3 28 days**	**SE-PA+ 1,25(OH)2-VD3 14 days**	**SE-PA+ 1,25(OH)2-VD3 28 days**
**IL4**	398.20 ± 85.04	383.77 ± 54.75	309.70 ± 62.34	282.48 ± 33.88	408.70 ± 74.81	291.98 ± 65.52	297.29 ± 90.03	308.18 ± 39.36	341.41 ± 57.87	256.28 ± 37.11	415.73 ± 82.64	309.24 ± 51.58
**IL10**	198.73 ± 23.78	209.08 ± 15.74	140.87 ± 17.86	134.65 ± 16.65	229.60 ± 13.55	206.67 ± 22.15	219.88 ± 17.49	166.79 ± 63.23	179.09 ± 14.43	130.13 ± 40.31	167.17 ± 6.57	140.28 ± 34.16
**IL13**	1268.54 ± 131.28	1502.71 ± 206.46	970.75 ± 50.11	889.97 ± 222.76	1496.25 ± 88.44	1424.69 ± 151.68	1406.60 ± 162.66	1205.62 ± 238.26	1205.30 ± 177.30	1001.83 ± 160.84	1170.47 ± 158.84	1080.52 ± 224.63
**IFNγ**	842.28 ± 112.93	405.01 ± 153.79	256.57 ± 124.10	958.48 ± 311.17	588.88 ± 176.93	744.95 ± 172.58	536.13 ± 156.59	972.04 ± 342.65	737.94 ± 91.64	1042.03 ± 250.25	724.43 ± 114.61	930.36 ± 153.49
**IL1β**	270.68 ± 42.25	530.58 ± 58.95	304.10 ± 44.67	238.70 ± 71.92	403.55 ± 50.29	228.46 ± 22.19	302.76 ± 56.44	297.59 ± 149.55	440.07 ± 64.58	290.63 ± 88.70	521.11 ± 72.01	425.26 ± 85.72
**IL6**	294.98 ± 19.55	374.12 ± 30.60	233.67 ± 68.09	254.99 ± 49.01	365.60 ± 93.10	319.18 ± 30.68	371.64 ± 55.75	357.66 ± 85.72	439.85 ± 40.23	348.09 ± 48.80	453.75 ± 22.58	403.48 ± 35.49
**IL12**	185.33 ± 22.75	105.44 ± 37.11	64.20 ± 25.66	167.59 ± 77.53	77.63 ± 43.19	133.16 ± 7.02	87.79 ± 31.17	169.23 ± 78.01	77.62 ± 25.94	223.50 ± 66.58	119.86 ± 13.21	299.58 ± 52.23
