# Supplementary figures and images for: Beneficial Impact of Inhaled 25(OH)-Vitamin D3 and 1,25(OH)2-Vitamin D3 on Pulmonary Response in the Murine Model of Hypersensitivity Pneumonitis
Source: Int J Mol Sci. 2024 Sep 24;25(19):10289. doi: 10.3390/ijms251910289 (PMC11476509; doi:10.3390/ijms251910289)

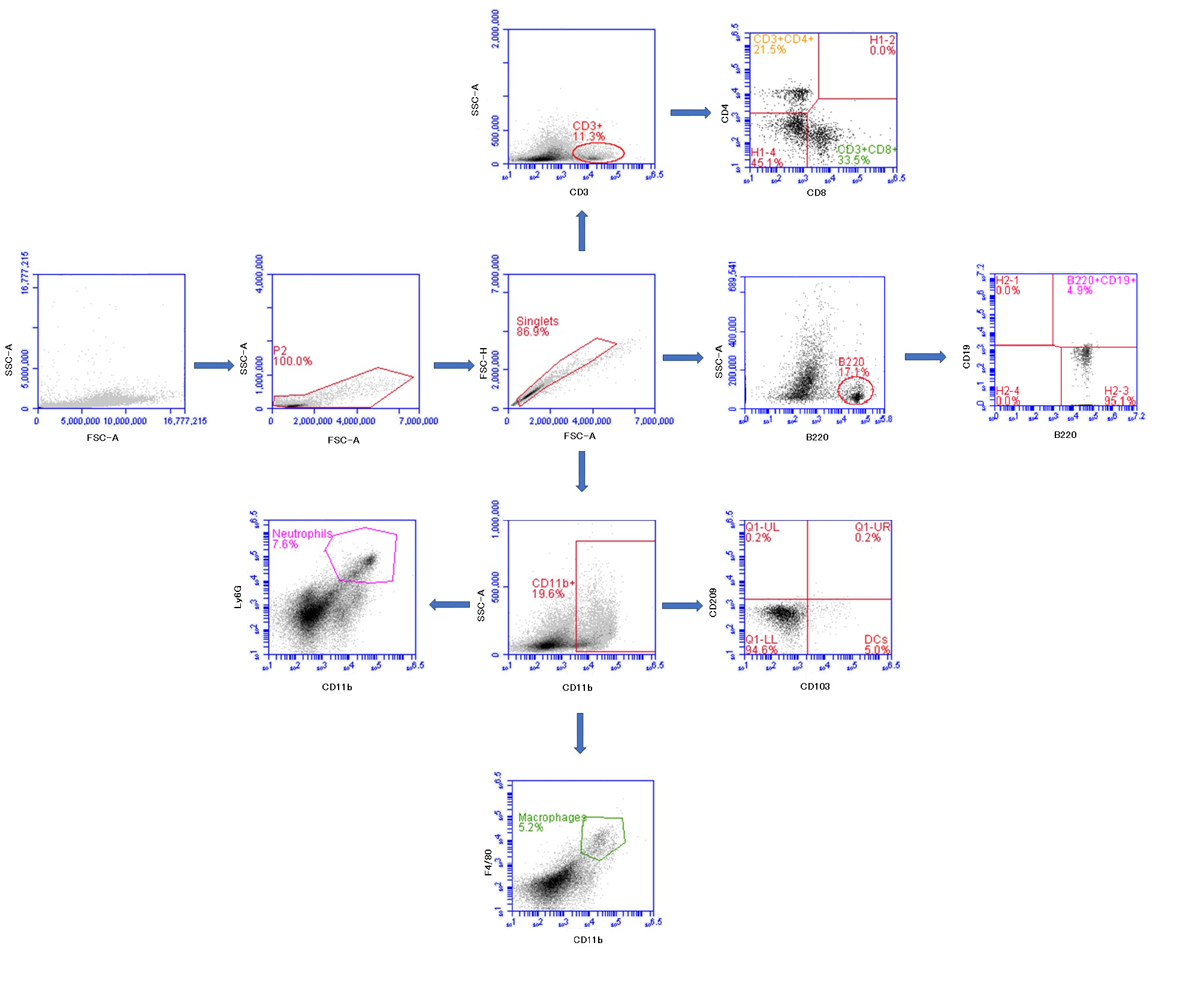

Supplement: Supplementary file 1 [file ijms-25-10289-s001.zip › Figure S1. Surface staining.tif]

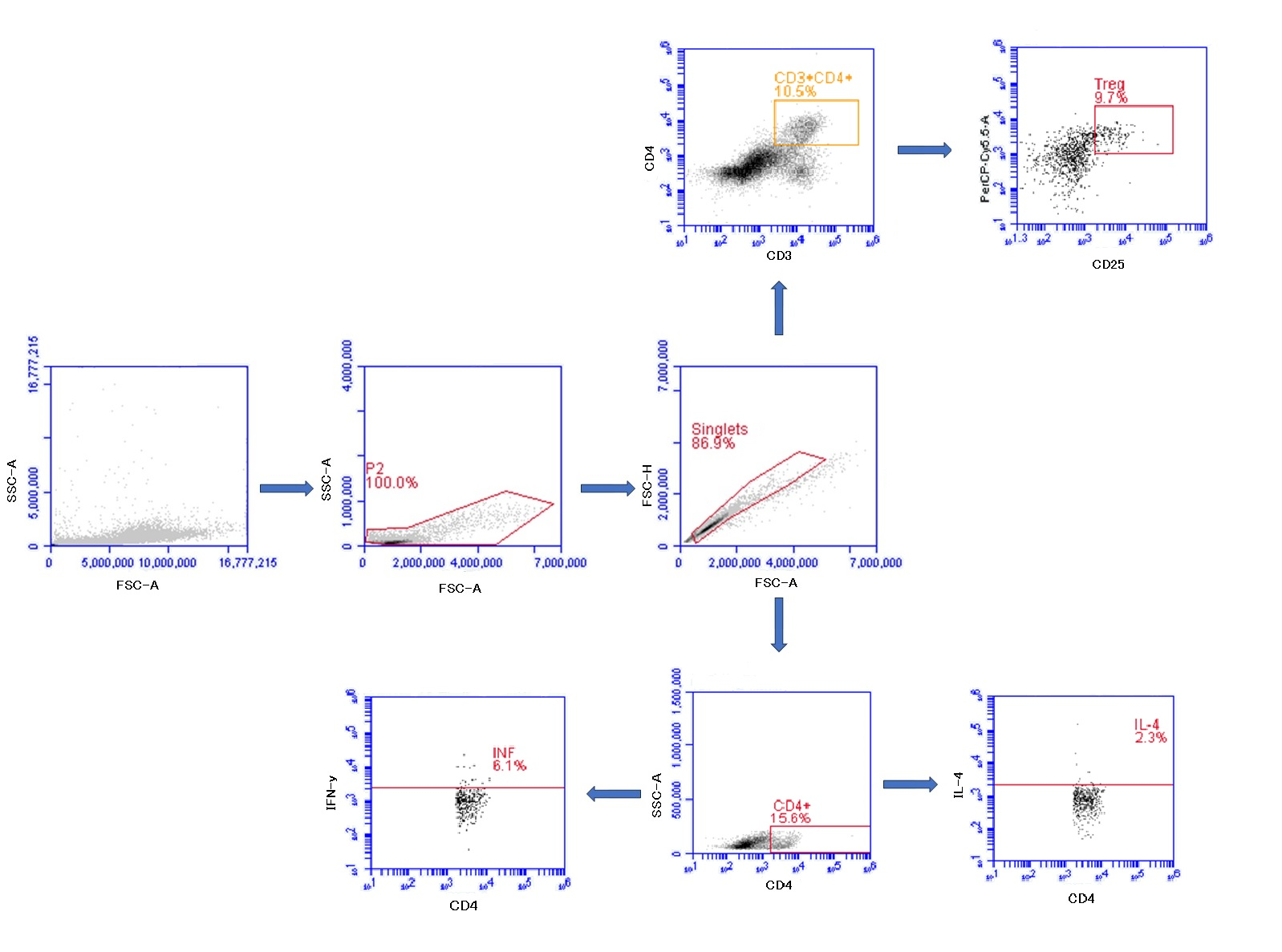

Supplement: Supplementary file 1 [file ijms-25-10289-s001.zip › Figure S2. Intracellular staining.tif]
